# Statistical learning to identify and characterise neurodevelopmental outcomes at 2 years in babies born preterm: model development and validation using population-level data from England and Wales

**DOI:** 10.1016/j.ebiom.2025.105811

**Published:** 2025-06-17

**Authors:** Sadia Haider, Athanasios Tsanas, G. David Batty, Rebecca M. Reynolds, Heather C. Whalley, Simon R. Cox, Riccardo E. Marioni, Cheryl Battersby, James P. Boardman

**Affiliations:** a Section of Neonatal Medicine, School of Public Health, Faculty of Medicine, Imperial College London, London, UK; b Usher Institute, Edinburgh Medical School, University of Edinburgh, Edinburgh, UK; c Alan Turing Institute, London, UK; d Department of Epidemiology and Public Health, University College London, London, UK; e Centre for Reproductive Health, Institute for Regeneration and Repair, University of Edinburgh, Edinburgh, UK; f Centre for Cardiovascular Science, University of Edinburgh, Edinburgh, UK; g Centre for Clinical Brain Sciences, University of Edinburgh, Edinburgh, UK; h Lothian Birth Cohorts, Department of Psychology, The University of Edinburgh, Edinburgh, UK; i Centre for Genomic and Experimental Medicine, Institute of Genetics and Cancer, University of Edinburgh, Edinburgh, UK

**Keywords:** Preterm, Neonatal, Neurodevelopmental impairments, Neurocognitive, Birth cohorts, Machine learning

## Abstract

**Background:**

Children born preterm face elevated risks of neurodevelopmental impairments across domains. Prior studies have relied on expert-imposed typologies within single domains. This study applies statistical learning to a national database to identify transdomain clusters and their maternal and neonatal predictors.

**Methods:**

Latent class analysis (LCA) was used to derive transdomain clusters from parent-reported visual, auditory, neuromotor, and communication impairments in preterm-born children at two years corrected age using the UK National Neonatal Research Database data (N = 27,261). Replication was conducted in an independent sample from Wales (N = 975). Clusters were clinically validated using cerebral palsy diagnosis, Bayley Scales of Infant and Toddler Development (3rd edition), and global neurodevelopmental delay. Random forest identified cluster-specific and shared predictors.

**Findings:**

Four homogeneous clusters were derived (silhouette score = 0.71) and replicated in Wales with high balanced accuracy (93%): (1) typically developing (84.8%), (2) communication impairments (8.4%), (3) neuro-motor impairments (4.1%), and (4) multiple neuro-morbidity (2.7%). Clusters had high clinical validity and were distinguishable by shared and cluster-specific predictors. Neonatal brain injuries were most predictive of neuro-motor and multiple neuro-morbidity clusters. Birthweight, gestational age, socio-economic deprivation, and sex were stronger predictors of the communication cluster than preterm co-morbidities.

**Interpretation:**

This study provides first evidence of the transdomain nature of neurodevelopmental impairments after preterm birth using LCA. The finding that socio-demographic and perinatal factors rather than co-morbidities increase the risk of communication impairment highlights the importance of environmental modification alongside clinical interventions. Applying data-driven approaches to routinely collected data may offer a cost-effective way to stratify at-risk children and inform targeted support strategies.

**Funding:**

10.13039/100014013UKRI Medical Research Council.

**Research in context**

**Evidence before this study**

We searched PubMed to October 8th, 2024, with no date or language restrictions for publications using the terms ((“preterm” OR “premature”) AND (“neurodevelopment” OR “neurocognitive”) AND (“cluster analysis” OR “machine learning”)). We defined outcomes *a priori* as singular domains (motor, language, neurocognitive). We identified twenty-nine results, which included two literature reviews on machine learning for the prediction of neurodevelopmental outcomes in preterm-born children before 3 years of age. There was no study using unsupervised cluster analysis from a national cohort of preterm-born children to investigate heterogeneity in comorbidity patterns across multiple domains of neurodevelopment.

**Added value of this study**

This is the first study to apply latent class analysis to routinely collected health data in England to derive unsupervised clusters of neurodevelopment at two years of age for children born preterm. Using parent-reported impairments across motor, communication, and neuro-sensory domains, we identified four novel, robust, and generalisable clusters of neurodevelopmental impairments: typically developing, communication impairments, neuromotor impairments, and multiple neuro-morbidity. These were replicated with high accuracy in a population-level cohort from Wales. The clusters were validated against clinical data and standardised assessments. Co-occurrence in multiple domains was common among children with impairments. Clusters were predicted by shared and specific maternal and neonatal features, which provide new insights into the ranked importance of perinatal variables for determining outcomes after preterm birth.

**Implications of all the available evidence**

Our study suggests that adverse neurodevelopmental outcomes are not inevitable for all preterm-born children by two years of age, although there are sub-groups of children at risk of developmental disadvantage before they start school. These findings could be clinically useful for identifying children requiring multidisciplinary support. Early and targeted interventions to reduce the risks associated with clusters are needed to optimise functioning across the life course. Longer-term follow-up is required to ascertain if impaired functioning manifests later in school age and the impact on educational outcomes. Routinely collected parent-reported outcomes in the National Neonatal Research Database (NNRD) may be a cost-effective data-source for identifying at-risk groups.

## Introduction

Preterm birth (PTB), defined as birth at less than 37 weeks of gestation, affects over 13 million pregnancies globally per annum.^1^ Over the past two decades, the survival rate of children born preterm has improved due to advances in perinatal medicine; however, neurodevelopmental impairment (NDI) among survivors is common: 10–15% of children born very preterm (<32 weeks) develop cerebral palsy and 30–50% have cognitive impairment and social and emotional difficulties.^2^ Preterm birth accounts for one of the highest numbers of disability-adjusted life years of any single childhood condition.^2^^,^^3^

Researchers often rely on typologies assigned by practitioners to categorise NDIs, which label outcomes using arbitrary thresholds to differentiate typical from atypical development.^4^^,^^5^ A limitation of investigator-imposed typologies is their inability to capture the extent of co-occurring impairments across diagnostic groups, the number and severity of which may have different impacts on functioning and quality of life.

To overcome the limitations of traditional diagnostic boundaries for conceptualising NDIs, transdiagnostic methods based on identifying shared mechanisms, processes, or brain structures that underlie NDIs have gained traction.^6^ The approach is enabled by data-driven methods that reveal shared neural, genetic, and behavioural mechanisms that span diagnoses. Transdiagnostic methods typically leverage complex multi-modal datasets, advanced computational techniques, and machine learning to identify latent structure that might not be visible using traditional, diagnosis-specific methods. More specifically, unsupervised machine learning techniques, such as cluster analysis, have been used to categorise individuals based on shared features (e.g., neuroimaging data, neurodevelopmental, or behavioural assessments) without relying on a priori diagnostic labels. Importantly, this can lead to the discovery of novel subtypes of patients who share similar profiles of impairments even if they have different diagnoses,^7^ and may provide new opportunities for investigating factors that predispose children to NDIs so informing interventions.

Here, we apply statistical learning methods to identify transdomain clusters in the National Neonatal Research Database (NNRD), which contains perinatal data and two-year outcome data for all infants born at less than 32 weeks cared for by the National Health Service (NHS) in the United Kingdom (UK). We test the following hypotheses. First, there are multiple transdomain clusters of NDIs in the preterm born population at two years corrected gestational age. Second, clusters have clinical validity. Third, clusters are predicted by maternal and neonatal clinical, demographic and social characteristics.

## Methods

### Study design and population

#### Data source

This retrospective cohort study uses data from the NNRD,^8^ a National Information Asset containing a standard data extract (the Neonatal Data Set, an NHS Information Standard; DAPB1595) from Electronic Patient Records (EPR). It includes 2-year outcomes for all babies admitted to neonatal units in England, Wales and Scotland. The NNRD contains data from approximately one million neonates from 2007 to the present, and contains approximately 450 de-identified entry items including demographic, diagnostic, daily, episodic, and outcome variables.^9^ All units in England and Wales have contributed data since 2012, and Scotland since 2018, providing whole population coverage for all preterm and sick infants admitted to neonatal units.^10^ Variable definitions are provided in Table 1.

**Table 1:** Definitions of variables used in the study.

Variable	Definition	Scale	Coding
***Maternal characteristics***			
Maternal ethnicity	Ethnicities were self-reported and grouped according to the 2011 National Census categories^11^		
White		Binary	1-0. Comparator group is all other ethnicities
	British		
	Irish		
	Irish traveller		
	Any other White backgrounds		
Mixed		Binary	1-0. Comparator group is all other ethnicities
	White and Black Caribbean		
	White and Black African		
	White and Asian		
	Any other mixed background		
Asian or Asian British		Binary	1-0. Comparator group is all other ethnicities
	Indian		
	Pakistani		
	Bangladeshi		
	Any other Asian background		
Black or Black British		Binary	1-0. Comparator group is all other ethnicities
	Caribbean		
	African		
	Any other Black background		
Other ethnic groups		Binary	1-0. Comparator group is all other ethnicities
	Chinese		
	Any other ethnic group		
	Not known		
IMD decile	Index of Multiple Deprivation score (IMD decile): the decile measuring the deprivation score for the mother's location of residence at the time of the infant's birth	Numeric	1–10. 1 being the most deprived and 10 being the least deprived
Maternal age	Age of mother at infant's birth	Numeric	
Smoking in pregnancy	Mother-confirmed smoking status during pregnancy	Binary	0 = Not smoking, 1 = Smoking
***Pre-existing maternal health conditions***			
Mental health		Binary	
Hypertension		Binary	
Diabetes		Binary	
***Maternal obstetric health problems***			
Maternal infection		Binary	
Maternal haemorrhage		Binary	
Prolonged rupture of membranes		Binary	
Gestational hypertension		Binary	
Gestational diabetes		Binary	
***Infant characteristics***			
Extent of prematurity	Derived from the categorisation of the number of completed weeks of gestation at birth	Categorical	0 = Very preterm (28–32 weeks), 1 = Extremely preterm (25–27 weeks), 2 = Limits of viability (22–24 weeks)
Birth-weight z-score	Weight z-scores were calculated using the LMS method of Cole and Green using sex, gestational age, postnatal age, and birth weight^12^, ^13^, ^14^	Numeric	
Multiple gestation	Derived from the number of foetuses at birth	Binary	0 = Singleton, 1 = Multiple
Sex	Biological sex assigned at birth	Binary	0 = Male, 1 = Female
Small for gestational age (SGA)	<10th centile for birth weight		
***Birth factors***			
Apgar 1 min	Apgar scores at 1 and 5 min: summary health score between 0 and 10 over 3 categories recorded for the infant at each indicated time from birth	Categorical	0 = Low (0–3), 1 = Intermediate (4–6), 2 = Normal (7–10)
Apgar 5 min	As above		0 = Low (0–3), 1 = Intermediate (4–6), 2 = Normal (7–10)
Vaginal delivery	Mother's mode of delivery	Binary	0 = C-section, 1 = Vaginal birth
***Neonatal comorbidities***			
Severe necrotising enterocolitis (NEC)	As milder forms of NEC are difficult to identify consistently and lack an agreed case-definition, we restricted ourselves to evaluating severe NEC. In accordance with the definition developed by Battersby et al.,^15^ severe NEC is defined as NEC that required surgery or was confirmed at death	Binary	0 = No severe NEC detected, 1 = Severe NEC present
Treated retinopathy of prematurity (ROP)	Whether a baby was surgically treated or given drugs (Avastin) for ROP	Binary	0 = No treatment given, 1 = Treatment given
Patent ductus arteriosus (PDA)	Whether surgical treatment was given for PDA	Binary	0 = No surgical treatment given for PDA, 1 = Surgical treatment given for PDA
Late onset sepsis	Bloodstream infection caused by pure growth of a clearly pathogenic organism ≥72 h of life	Binary	0 = No late onset sepsis detected, 1 = Late onset sepsis detected (pure pathogen growth)
Late blood growth (any)	Bloodstream infection caused by mixed growths or organisms of uncertain pathogenic significance (including skin commensals) ≥72 h of life	Binary	0 = No late blood growth detected, 1 = Late blood growth detected
***Neonatal brain injuries***			
Cystic periventricular leukomalacia (cPVL)	CPVL on cerebral ultrasound scan (CUSS) &/or in final diagnosis record	Binary	0 = No CPVL of no record of cPVL, 1 = cPVL detected
Porencephalic cyst	Porencephalic cyst visible on either the left or right hemispheres on CUSS &/or in final diagnosis record	Binary	0 = No porencephalic cyst of no record of porencephalic cyst, 1 = Porencephalic cyst detected
Hydrocephalus	Post-haemorrhagic hydrocephalus visible on CUSS &/or in final diagnosis record	Binary	0 = No hydrocephalus of no record of porencephalic cyst, 1 = Hydrocephalus detected
Intraventricular haemorrhage	The most severe grade of IVH seen on left/right hemisphere on the CUS scan &/or in final diagnosis record	Categorical	0 = No IVH, 1 = IVH grade 1 or 2, 2 = IVH grade 3 or 4
***Interventions***			
Ante-natal steroids	Whether a complete course of antenatal steroid courses given during pregnancy	Binary	0 = None or incomplete course, 1 = Complete course
Surfactant at resuscitation	Surfactant given at resuscitation	Binary	0 = No, 1 = Yes
Invasive respiratory support	Non-invasive respiratory support received during admission to NNU	Binary	0 = No, 1 = Yes
Non-invasive respiratory support	Invasive respiratory support received during admission to NNU	Binary	0 = No, 1 = Yes
Post-natal steroids	Post-natal steroids received by 36 weeks' post-menstrual age	Binary	0 = No, 1 = yes
Antibiotics	Antibiotics received by 36 weeks' post-menstrual age	Binary	0 = No, 1 = Yes
***Discharge***			
Discharged on oxygen	Discharged with home oxygen therapy	Binary	0 = No, 1 = Yes
Discharged on breast milk	Discharged on breast milk versus no milk, formula, mixed, or other feeding.	Binary	0 = No, 1 = Yes
Discharge destination	Discharged home versus to a ward or hospital	Binary	0 = Ward/hospital, 1 = Home
**Year-two outcomes**			
***Neuro-sensory***			
**Visual impairment**	No impairment	Binary	0 = No visual impairment, 1 = At least 1 positive response to impairment-specific question
	Does this child have any visual problems (including squint)? (Mild/moderate)		
	Does this child have visual defect that is not fully correctable? (Mild/moderate)		
	Is this child blind or sees light only? (Severe)		
**Auditory impairment**			
	No impairment	Binary	0 = No auditory impairment, 1 = At least 1 positive response to impairment-specific question
	Does this child have a hearing impairment?		
	Does this child have hearing impairment corrected by aids? (Mild/moderate)		
	Does this child have hearing impairment not correctable with aids? (Severe)		
***Gross motor***			
**Walking impairment**		Binary	0 = No walking impairment, 1 = At least 1 positive response to impairment-specific question
	No impairment		
	Does this child have any difficulty walking?		
	Is this child's gait non-fluent or abnormal reducing mobility? (Mild/moderate)		
	Is this child unable to walk without assistance? (Severe)		
**Sitting impairment**		Binary	0 = No sitting impairment, 1 = At least 1 positive response to impairment-specific question
	No impairment		
	Is this child unstable or needs to be supported when sitting? (Mild/moderate)		
	Is this child unable to sit? (Severe)		
***Fine motor***			
**Hand impairment**		Binary	0 = No hand impairment, 1 = At least 1 positive response to impairment-specific question
	No impairment		
	Does this child have any difficulty with the use of one hand? (Mild/moderate)		
	Does this child have difficulty with the use of both hands? (Mild/moderate)		
	Is this child unable to use hands (i.e., to feed)? (Severe)		
***Communication***			
**Comprehension/receptive language impairment**		Binary	0 = No comprehension impairment, 1 = At least 1 positive response to impairment-specific question
	No impairment		
	Does this child have difficulty with understanding words or signs outside of familiar context? (Mild/moderate)		
	Is this child unable to understand words or signs? (Severe)		
**Speech/expressive language impairment**		Binary	0 = No speech impairment, 1 = At least 1 positive response to impairment-specific question
	No impairment		
	Does this child have difficulty with speech (<10 words/signs)? (Mild/moderate)		
	Does the child have <5 meaningful words, vocalisations or signs? (Severe)		
**Cerebral palsy**	Parent report of clinical diagnosis	Binary	0 = No CP, 1 = Diagnosed CP
**Cerebral palsy type**	Parent report of clinical diagnosis of the type of cerebral palsy	Categorical	0 = No CP, 1 = Dyskinetic/Dystonic/Choreo-athetoid, 2 = Hemiplegia left or right side, 3 = Spastic bilateral: 2 limbs, 4 = Spastic bilateral: 3 limbs, 5 = Spastic bilateral: 4 limbs, 6 = Not classifiable
**Developmental delay**	Healthcare professional global assessment of developmental delay at 2-year follow-up		
Typically developing	<3 months delay	Categorical	0 = Typically developing, 1 = Mild delay, 2 = Moderate delay, 3 = Severe delay
Mild delay	3–6 months delay		
Moderate delay	6–12 months delay		
Severe delay	>12 months delay		
**Bayley scales of toddler & infant development (third edition) composite scores**^16^			
Language	Derived from expressive and receptive communication subtests	Numeric	All three composite scores are age-standardised with a mean (SD) score of 100
Cognitive	Derived from the cognition subtest		
Motor	Derived from fine and gross motor subtests		

For the year-two outcomes, impairment-specific questions were assigned a label of “no”, “mild/moderate” or “severe” impairment as reported in the NNRD study by Wong, Cowan, & Modi.^17^

#### Assessment at two years of corrected age

Neurodevelopmental data in the NNRD were obtained from clinician-entered EPR. There are four sources of data: (1) Thames Regional Perinatal Outcome Group-Standard Electronic Neonatal Database–National Neonatal Audit Programme (TRPG-SEND-NNAP) questionnaire, a tool used across the UK to collect parent-reported neurodevelopmental functions, (2) clinical diagnoses of cerebral palsy,^18^ (3) Health Care Practitioner (HCP) assessment of global developmental delay based on the perceived number of months of delay (typically developing (<3 months of delay), mild delay (3–6 months), moderate delay (6–12 months), and severe delay (>12 months)), and (4) standardised assessments using validated tools, most commonly the Bayley Scales of Infant and Toddler Development 3rd edition (BSID-III).^19^

#### Extract and inclusion criteria

We extracted patient-level data for all infants born before 32 completed weeks gestation between 1st January 2007 and 31st December 2019 who were admitted to NHS neonatal units in England and Wales, survived to two-years, and had non-missing information on all eight parent-reported impairments. Babies with congenital malformations at birth, missing data for gestational age, sex, and place of birth were excluded.

### Ethics

Ethical and regulatory approval has been granted to use de-identified data from the NNRD without informed consent as part of the neoWonder study (East Midlands-Leicester South Research Ethics Committee reference 21/EM/0130).^20^ Neonatal units contributing to the NNRD were informed through their UK Neonatal Collaborative lead about the study, no opt-out requests were received. Data from Scotland were not accessible under the governance arrangements for this study.

### Primary outcome: two-year neurodevelopment

At two-year follow-up, parents reported their child's neurodevelopmental functioning using the TRPG-SEND-NNAP questionnaire.^21^ These include questions about function: neuro-sensory (auditory and visual), fine motor (hand), gross motor (sitting and walking), and communication (speech and comprehension) impairments. For each impairment, we created a binary variable where ‘0’ denoted no impairment and ‘1’ if there was a positive response to any impairment-related question. Additionally, parents reported whether a clinical diagnosis of cerebral palsy (CP) was made.

### Predictor variables: maternal and neonatal characteristics

For estimating cluster-specific associations with maternal and neonatal characteristics, we selected 43 variables of confirmed or putative importance for neurodevelopment, including maternal and infant demographics, pre-existing and gestational maternal health problems, birth factors, co-morbidities of preterm birth, brain injuries, medical exposures, and characteristics at discharge (Table 1).^22^

### Statistics

#### Descriptive analyses

We report patient characteristics for the study cohort by birth in England (development cohort) and Wales (validation cohort). To test for differences in categorical variables between England and Wales, Chi-square tests were used. The Shapiro–Wilk test was applied to continuous variables to assess whether they conformed to normal distributions prior to applying non-parametric Wilcoxon rank sum tests. A significance value threshold of p < 0.05 was specified.

### Latent class analysis

We used LCA to investigate heterogeneity in patterns of neurodevelopment, with the aim of identifying distinct subgroups in multivariate categorical data that share similar response patterns to the impairment indicators.^23^ Unlike traditional deterministic clustering methods, for example, the standard k-means, LCA is model-based, allowing for probabilistic assignment of individuals to clusters. Each child is assigned to the cluster where they have the highest posterior probability of belonging, rather than by strict assignment of individuals into clusters. To assess generalisability, LCA was first conducted on England data using the eight impairments, then validated on Wales data.

To determine the preferred number of clusters, models with 1–6 clusters were estimated and compared using (i) elbow plots of Bayesian Information Criterion (BIC), sample-size adjusted BIC (aBIC), and Akaike Information Criterion (AIC),^24^ where lower values indicate better fit and (ii) bootstrapped likelihood ratio tests (BLRT),^25^ where a significant p-value (p < 0.05) suggests better fit of a k-cluster model over a k−1-cluster model. When criteria conflicted, elbow plots were prioritised.

Latent class analysis (LCA) assumes local independence, i.e., indicator variables are conditionally independent given class membership. This was assessed using bivariate residuals (BVR), where a value >4 indicates significant residual association unexplained by the classes.^26^ Classification certainty was evaluated through average posterior probabilities,^24^ with values closer to 1 indicating greater confidence. The average silhouette width (ASW) was calculated to examine how well each observation fits into its assigned cluster and the degree of separation between them. Cluster labels were informed by plots of impairment prevalence within each cluster and clinical interpretation. Tetrachoric correlation analysis explored associations between impairments and cluster membership, with coefficients >|0.7| considered strong. Stability of the LCA solution was tested using 500 replications with random starting values. Model estimation used the expectation-maximisation algorithm and was conducted in Mplus version 8.0.2.^27^

#### Clinical validation of clusters

LCA clusters were validated by assessing agreement with standardised assessment and clinical data not included in the clustering: (1) cerebral palsy type, (2) motor, language, and cognitive scores from the BSID-III, (3) HCP assessment of global delay developmental delay in months. Agreement of the clusters with cerebral palsy type was assessed by plotting the prevalence of CP type by clusters. For BSID-III scores, we compared mean and 95% CI scores. Agreement with HCP assessment was ascertained by calculating the Adjusted Rand Index and visualisation using an alluvial plot.

### Visualisation of cluster structure

To visualise the cluster structure, the t-Distributed Stochastic Neighbour Embedding (t-SNE) algorithm was applied, projecting the 8-dimensional data onto a 2-dimensional plot.^28^ Given that there is no underlying ground truth to compare the LCA solution against, t-SNE plots enabled us to assess how well the clusters were separated in the projected two-dimensional feature space by colour mapping each observation with the class labels and each impairment feature. Grid search was used to select the perplexity and theta hyper-parameters with values ranging from 5 to 300 for perplexity and values of 0.1, 0.3, and 0.5 for theta.^29^ Optimal hyper-parameters were selected by visual inspection of the scatter-plots. “Rtsne” and “ggplot2” R packages were used to generate the t-SNE plots.

#### Random forest for cluster validation

A Random Forest (RF) model was trained on the England cohort to predict cluster labels in the Wales cohort. The agreement between observed clusters (those determined by LCA) and those predicted in the Wales cohort by training a RF on England data was evaluated using a confusion matrix and balanced accuracy score. To further assess the generalisability of the results, this analysis was also conducted by reversing the training and testing datasets.

#### Cluster stability

To evaluate cluster stability across cohorts, the adjusted Rand index (ARI)^30^ was calculated to evaluate the agreement between individual assignments when LCA was applied to each cohort separately compared to when the data was pooled and modelled jointly. A value of 1 indicates perfect agreement and a value close to 0 indicates that the two partitions are no better than random assignments.

### Estimation of cluster-specific ante- and peri-natal features

#### Imputation of missing data in the training set

We used non-parametric imputation by chained random forest for imputing missing values of continuous and categorical predictors.^31^ Briefly, each variable is imputed by predictions from a random forest using all other predictor variables as auxiliary variables. The algorithm iterates multiple times over all variables, improving the accuracy of the imputations with each iteration until the average out-of-bag prediction error of the models stabilises. We specified 100 iterations. We used the “missRanger” R package for imputation.^31^

#### Missing data in the test set

To handle of missing data on the features in the test set, we applied mode imputation for categorical predictors and median imputation for continuous features. The mode/median of each variable was calculated in the training dataset (England cohort), and missing values in the test set (Wales cohort) were replaced with the corresponding values.

#### Feature selection

We utilised the Boruta algorithm to select important features prior to prediction using RF. Boruta algorithm is a wrapper feature selection technique built around the RF learner, which compares feature importance (z-scores) to shuffled shadow features, retaining those that consistently outperform shadows. Underperforming features are removed. We used the “Boruta” R package for feature selection.^32^

#### Random forest estimation of cluster-specific ante- and peri-natal features

For the subset of features identified from the feature selection process, a multi-class RF with 10-fold cross-validation was used to identify the most important features for prediction of each cluster. After training the models on England data, the Wales cohort was used as the test set to assess model performance using the confusion matrix of observed and predicted classes and computing the balanced accuracy score. Model fitting was carried out in R using “RandomForest” and “Caret” R packages.^33^

#### Class imbalance

The data set was imbalanced since 85% of the cohort was classified as typically developing. To overcome this, we explored different data balancing techniques: under-sampling of the majority classes to match the number in the minority class, class weights that are inversely proportional to class frequencies and synthetic minority over-sampling technique (SMOTE).^34^ This is a training data enrichment method where we oversampled from the minority class to match the number of samples from the majority class by creating new synthetic samples to create a balanced data set. The “themis” package in R was used for under-sampling and SMOTE of the cluster.

#### Explainability and SHAP analysis

SHapley Additive exPlanations (SHAP) analysis was used to provide insights into the contribution of each feature to the cluster predictions.^35^ SHAP is a unified framework that extends the classic Shapley values from game theory to explain the output of “black box” machine learning models.^35^ We visualised the global importance of the features using a bar plot where the bars show in descending order of importance the impact each feature has on the model predicting each cluster. SHAP dependence plots were used to visualise how changes in a feature's value influence its contribution to the model's prediction. The “shapviz” package in R was used calculation and plotting of SHAP values.^36^

#### Sensitivity of RF results to missing data on features

To investigate RF sensitivity to imputation, we compared feature prevalence in complete and incomplete samples. We then ran 10-fold cross-validated RF models using complete data and repeated this for observations with imputed data. For both models, RF importance scores were normalised by the maximum importance score, and differences for each feature plotted. Balanced accuracy was compared when models were tested on unseen (Wales) data.

**Fig. 1:**
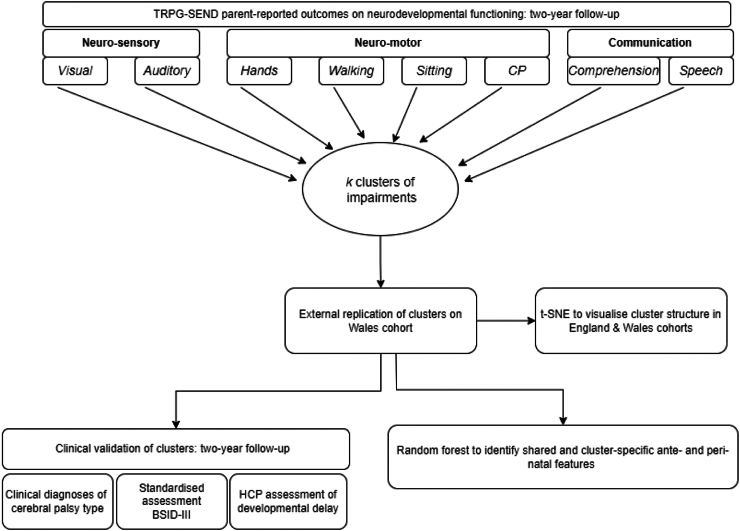
**Overview of analytic steps**. LCA was used to derive transdomain clusters of neurodevelopmental functioning using unvalidated parent-reported outcomes in the England cohort. Clusters were externally replicated using the Wales cohort. t-SNE algorithm was used to compare cluster structure in both cohorts. Clusters were clinically validated using three additional sources of data at the two-year follow-up, and RF was used to investigate shared and cluster-specific ante- and peri-natal features.

Fig. 1 presents an overview of the analytic approach.

### Role of funders

The funder of the study had no role in study design, data collection, data analysis, data interpretation, or writing of the report.

## Results

### Participant flow

27,261/31,042 (88%) children in the England cohort and 975/1098 (89%) in the Wales cohort with complete information on all impairments were included in the analysis (Figure S1).

### Characteristics of the study population

Table 2 shows participant characteristics. In the England cohort, 53.3% were male, with no sex differences between the cohorts. Sixty-one percent (61.1%) were very preterm (born 28–32 weeks), 32% extremely preterm (25–27 weeks), and 6.9% at 22–24 weeks. 93.7% of children in Wales were born to White mothers compared with 72.7% in England. Data completeness for two-year outcomes improved over time.^10^^,^^37^

**Table 2:** Selected characteristics of the study population.

Variable	England	Wales	Total	p-value
N	27,261 (96.5%)	975 (3.5%)	28,236 (100.0%)	
**Maternal age**	30.9 (6.2)	29.4 (5.8)	30.8 (6.2)	<0.0001
**Corrected gestational age at 2-year follow-up (years)**	2.1 (0.22)	2.1 (0.16)	2.1 (0.22)	0.017
**Birth year**				<0.0001
2007–2011	6593 (24.2)	11 (1.1)	6604 (23.4)	
2012–2014	10,843 (39.8)	424 (43.5)	11,267 (39.9)	
2015–2019	9825 (36)	540 (55.4)	10,365 (36.7)	
**Mother's ethnicity**				<0.0001
White	17,767 (72.7%)	679 (93.7%)	18,446 (73.3%)	
Asian	3292 (13.5%)	22 (3.0%)	3314 (13.2%)	
Black	2385 (9.8%)	11 (1.5%)	2396 (9.5%)	
Mixed	434 (1.8%)	7 (1.0%)	441 (1.8%)	
Other	551 (2.3%)	6 (0.8%)	557 (2.2%)	
**IMD decile**				0.044
1–most deprived	4440 (16.9%)	146 (15.3%)	4586 (16.8%)	
2	3689 (14.0%)	144 (15.1%)	3833 (14.1%)	
3	3207 (12.2%)	91 (9.5%)	3298 (12.1%)	
4	2791 (10.6%)	119 (12.4%)	2910 (10.7%)	
5	2505 (9.5%)	106 (11.1%)	2611 (9.6%)	
6	2234 (8.5%)	86 (9.0%)	2320 (8.5%)	
7	2097 (8.0%)	63 (6.6%)	2160 (7.9%)	
8	1953 (7.4%)	72 (7.5%)	2025 (7.4%)	
9	1817 (6.9%)	62 (6.5%)	1879 (6.9%)	
10–least deprived	1563 (5.9%)	67 (7.0%)	1630 (6.0%)	
**Maternal smoking**				<0.0001
No	23,105 (84.8%)	763 (78.3%)	23,868 (84.5%)	
Yes	4156 (15.2%)	212 (21.7%)	4368 (15.5%)	
**Birthweight z-score**	−0.37 (0.89)	−0.26 (0.88)	−0.36 (0.89)	<0.0001
**Gestational age at birth (median age (weeks), IQR)**	29 (27–30)	28 (26–29)	28 (26–29)	<0.0001
**Sex assigned at birth**				0.087
Male	14,535 (53.3%)	547 (56.1%)	15,082 (53.4%)	
Female	12,726 (46.7%)	428 (43.9%)	13,154 (46.6%)	
**Degree of prematurity (gestational age (weeks))**				<0.0001
Very preterm (28–32 weeks)	16,645 (61.1%)	674 (69.1%)	17,319 (61.3%)	
Extremely preterm (25–27 weeks)	8736 (32.0%)	248 (25.4%)	8984 (31.8%)	
Limits of viability (22–24 weeks)	1880 (6.9%)	53 (5.4%)	1933 (6.8%)	
**Multiple birth**				0.103
Singleton	19,877 (72.9%)	734 (75.3%)	20,611 (73.0%)	
Multiple	7381 (27.1%)	241 (24.7%)	7622 (27.0%)	
**Severe NEC**				0.008
No	26,553 (97.4%)	963 (98.8%)	27,516 (97.5%)	
Yes	708 (2.6%)	12 (1.2%)	720 (2.5%)	
**Treated ROP**				0.475
No	25,934 (95.2%)	923 (94.7%)	26,857 (95.1%)	
Yes	1317 (4.8%)	52 (5.3%)	1369 (4.9%)	
**Late onset sepsis**				<0.0001
No	26,152 (95.9%)	911 (93.4%)	27,063 (95.8%)	
Yes	1109 (4.1%)	64 (6.6%)	1173 (4.2%)	
**Cystic periventricular leukomalacia (cPVL)**				0.500
No	26,703 (98.0%)	952 (97.6%)	27,655 (97.9%)	
Yes	558 (2.0%)	23 (2.4%)	581 (2.1%)	
**Intraventricular haemorrhage (IVH)**				0.487
No	21,851 (80.2%)	771 (79.1%)	22,622 (80.1%)	
Grade 1 or 2	4131 (15.2%)	161 (16.5%)	4292 (15.2%)	
Grade 3 or 4	1279 (4.7%)	43 (4.4%)	1322 (4.7%)	
**Porencephalic cyst**				0.448
No	26,790 (98.3%)	955 (97.9%)	27,745 (98.3%)	
Yes	471 (1.7%)	20 (2.1%)	491 (1.7%)	
**Hydrocephalus**				0.808
No	26,786 (98.3%)	957 (98.2%)	27,743 (98.3%)	
Yes	475 (1.7%)	18 (1.8%)	493 (1.7%)	

p-values represent Chi-square differences in categorical variables or Wilcoxon rank sum tests for continuous variables for differences between England and Wales. A threshold of <0.05 indicates significant differences.

Frequencies (%) and mean (SD) are presented for categorical and continuous variables, respectively.

Two-year neurodevelopmental outcomes for the England and Wales cohorts are shown in Table 3 There were no significant differences in the prevalence of parent-reported or HCP-reported impairments between the two countries.

**Table 3:** The distribution of neuro-developmental outcomes in England and Wales for children with a record of a TRPG-SEND assessment at two years corrected gestational age.

Impairment (Chi-square p-value England v Wales)	England freq. (%)	Wales freq. (%)	Total freq. (%)
N	32,384 (96.6%)	1149 (3.4%)	33,533 (100.0%)
**Gestational age (weeks) (p<0.0001)**			
<30 weeks	24,799 (76.6%)	699 (60.8%)	25,498 (76%)
30 or 31 weeks	7585 (23.4%)	450 (39.2%)	8035 (24%)

**Parent-reported outcomes on neurodevelopmental functioning**			
**Neuro-sensory**			
**Visual (p=0.181)**			
No impairment	26,921 (90.9%)	974 (92.7%)	27,895 (91.0%)
Does this child have any visual problems (including squint)?	2175 (7.3%)	64 (6.1%)	2239 (7.3%)
Does this child have visual defect that is not fully correctable?	411 (1.4%)	12 (1.1%)	423 (1.4%)
Is this child blind or sees light only?	107 (0.4%)	1 (0.1%)	108 (0.4%)
**Auditory (p=0.283)**			
No impairment	28,781 (96.7%)	1036 (97.7%)	29,817 (96.7%)
Does this child have a hearing impairment?	420 (1.4%)	10 (0.9%)	430 (1.4%)
Does this child have hearing impairment corrected by aids?	361 (1.2%)	10 (0.9%)	371 (1.2%)
Does this child have hearing impairment not correctable with aids?	215 (0.7%)	4 (0.4%)	219 (0.7%)
**Gross motor**			
**Walking (p=0.073)**			
No impairment	26,897 (87.9%)	984 (90.2%)	27,881 (87.9%)
Does this child have any difficulty walking?	290 (0.9%)	11 (1.0%)	301 (0.9%)
Is this child's gait non-fluent or abnormal reducing mobility?	1230 (4.0%)	29 (2.7%)	1259 (4.0%)
Is this child unable to walk without assistance?	2198 (7.2%)	67 (6.1%)	2265 (7.1%)
**Sitting (p=0.561)**			
No impairment	29,426 (96.9%)	1064 (97.3%)	30,490 (96.9%)
Is this child unstable or needs to be supported when sitting?	393 (1.3%)	14 (1.3%)	407 (1.3%)
Is this child unable to sit?	561 (1.8%)	15 (1.4%)	576 (1.8%)
**Fine motor**			
**Hands (0.840)**			
No impairment	28,717 (94.7%)	1038 (95.1%)	29,755 (94.7%)
Does this child have any difficulty with the use of one hand?	531 (1.8%)	16 (1.5%)	547 (1.7%)
Does this child have difficulty with the use of both hands?	331 (1.1%)	13 (1.2%)	344 (1.1%)
Is this child unable to use hands (i.e., to feed)?	748 (2.5%)	24 (2.2%)	772 (2.5%)
**Communication**			
**Comprehension (p=0.257)**			
No impairment	27,448 (93.1%)	973 (92.1%)	28,421 (93.0%)
Does this child have difficulty with understanding words or signs outside of familiar context?	964 (3.3%)	35 (3.3%)	999 (3.3%)
Is this child unable to understand words or signs?	1086 (3.7%)	49 (4.6%)	1135 (3.7%)
**Speech (p=0.159)**			
No impairment	22,561 (75.3%)	787 (73.5%)	23,348 (75.3%)
Does this child have difficulty with speech (<10 words/signs)?	3731 (12.5%)	154 (14.4%)	3885 (12.5%)
Does the child have <5 meaningful words, vocalisations or signs?	3650 (12.2%)	130 (12.1%)	3780 (12.2%)

**Healthcare-professional diagnoses**			
**Cerebral palsy (parent-report of clinical diagnosis) (p = 0.175)**			
No	27,882 (93.8%)	976 (94.8%)	28,858 (93.8%)
Yes	1851 (6.2%)	53 (5.2%)	1904 (6.2%)
**Healthcare-professional assessment of developmental delay (months of delay) (p<0.0001)**			
Typically developing (<3 months)	20,983 (72.3%)	656 (65.6%)	21,639 (72.1%)
Mild delay (3–6 months)	3788 (13.1%)	157 (15.7%)	3945 (13.1%)
Moderate delay (6–12 months)	2586 (8.9%)	119 (11.9%)	2705 (9.0%)
Severe delay (>12 months)	1651 (5.7%)	68 (6.8%)	1719 (5.7%)

p-values represent Chi-square differences in impairment distributions between England and Wales.

### Identification of clusters of neuro-developmental impairments using LCA

#### Identifying the number of clusters

We applied LCA on 27,261 children from the England cohort and 975 from the Wales cohort. For the England cohort, the elbow plot of information criteria (IC) shows little improvement in fit between four and six clusters. For Wales, the lowest IC values were obtained for the four-cluster model, and there was an increase thereafter (Table S1, Figure S2).

Figure S3 shows the evolution of clusters across models with each additional cluster for England and Wales separately. In addition to the minimal improvements to fit, clinical interpretation of each sub-group beyond the four-cluster model was non-trivial. For reasons of parsimony, the four-cluster model was chosen as the preferred solution.

Average posterior assignment probabilities, computed independently for the two cohorts, were high for all clusters and ranged from 0.87 to 0.94 (Table 4, Table 5).

**Table 4:** Average latent posterior probability of the class membership (row) conditional on most likely class assignment (column) in England.

The posterior probabilities in the diagonal cells represent the average probability of a child being assigned to a class given their observed response patterns for variables in the model. Diagonal values approaching 1 and off-diagonal values approaching 0 indicate good classification quality, thus, individuals are well-classified in the most likely class and have low probabilities of being assigned to other groups. The largest probabilities for alternative class membership were between MNM and NM (0.097) and COMM and TD (0.087).

TD = Typically developing, COMM = Communication impairments, NM = Neuro-motor impairments, MNM = Multiple neuro-morbidity.

**Table 5:** Average latent posterior probability of the class membership (row) conditional on most likely class assignment (column) in Wales.

The posterior probabilities in the diagonal cells represent the average probability of a child being assigned to a class given their observed response patterns for variables in the model. Diagonal values approaching 1 and off-diagonal values approaching 0 indicate good classification quality, thus, individuals are well-classified in the most likely class and have low probabilities of being assigned to other groups. The largest probabilities for alternative class membership were between MNM and NM (0.097) and COMM and TD (0.087).

TD = Typically developing, COMM = Communication impairments, NM = Neuro-motor impairments, MNM = Multiple neuro-morbidity.

Table S2 shows that no bivariate residual exceeded the threshold of four, indicating that the four-class model adequately accounted for correlations among the impairments and that the assumption of local independence was met.

#### Description of the clusters

**Fig. 2:**
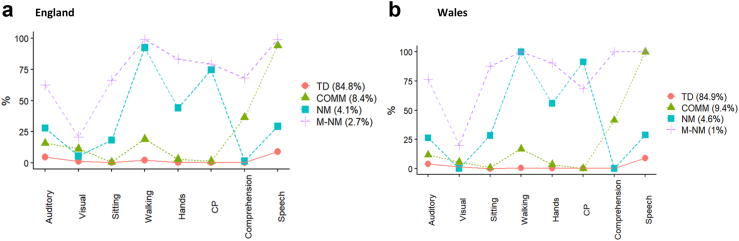
**Four clusters identified by latent class analysis in 27,261 children ina)England and 975 children inb)Wales at 2-years of age**. The children were assigned to each cluster according to the maximum posterior probability. The lines display the prevalence of each impairment conditional upon cluster membership. TD = Typically developing, COMM = Communication impairments, NM = Neuro-motor impairments, MNM = Multiple neuro-morbidity.

Fig. 2 shows the proportion of children assigned to each cluster and the prevalence of impairments within each. Table 6, Table 7 show descriptive statistics and the tetrachoric correlation coefficients of impairments with each cluster. Identified clusters were labelled according to predominant features as i) typically developing (TD), ii) communication impairments (COMM), iii) neuro-motor impairments (NM), and iv) multiple neuro-morbidity (MNM). For England, the clusters were characterised as follows:

**Table 6:** Correlation of impairments with clusters.

Tetrachoric correlation coefficients for binary data. Clusters were one-hot encoded to binary variables.

For developmental delay (as diagnosed by a HCP), typically developing: <3 months delay; mild delay: 3–6 months delay; moderate delay: 6–12 months delay; severe delay: >12 months delay.

TD = Typically developing, COMM = Communication impairments, NM = Neuro-motor impairments, MNM = Multiple neuro-morbidity.

**Table 7:** Correlation of impairments with HCP assessment of developmental delay.

Tetrachoric correlation coefficients for binary data. Clusters were one-hot encoded to binary variables.

For developmental delay (as diagnosed by a HCP), typically developing: <3 months delay; mild delay: 3–6 months delay; moderate delay: 6–12 months delay; severe delay: >12 months delay.

TD = Typically developing, COMM = Communication impairments, NM = Neuro-motor impairments, MNM = Multiple neuro-morbidity.

*Typically developing* (84.8% of children): children had a low risk of impairments. 79.5% had no impairment and 20.2% had a single impairment, the most frequent being mild speech difficulty. Each impairment was strongly negatively correlated with cluster membership.

*Communication* (8.4%): Most children were reported by their parents to have speech impairment (94.2%) and a third was reported to have comprehension difficulty (36.5%). The risk of other impairments was relatively low (<25%). Neuro-motor impairments (walking, hands, and CP) were negatively correlated with this cluster.

*Neuro-motor* (4.1%): Children were characterised by a high risk of gross and fine motor impairments (walking 93%, hands 44%). 74% of children had CP. Whilst 29% of children had a reported speech difficulty, comprehension impairments were absent.

*Multiple neuro-morbidity* (2.7%): This was the smallest cluster, characterised by the highest prevalence, number of, and co-occurrence of impairments. A similar proportion of children had CP in NM and MNM, however, in contrast to the former, most children in the MNM had reported comprehension difficulties (68%).

The finding that 85% of children were assigned to the typically developing cluster must be interpreted with caution. It is possible that our study has not captured the full breadth of impairments at two-years of age (e.g., behavioural, autism spectrum disorder (ASD), social communication) or that impairments may manifest at a later age.^38^

### External validation

To assess how well the LCA methodology generalised between the two cohorts, we trained a RF model using the development dataset (England) and assessed performance on the validation dataset (Wales). We achieved a 93% balanced accuracy when we predicted cluster labels on unseen Wales data after training a random forest model on the England cohort with the 8 features as inputs and cluster labels as outputs. To further validate the robustness of the methodology, we reversed the training and testing datasets and a similar performance was achieved (Figure S6).

### t-SNE visualisations

**Fig. 3:**
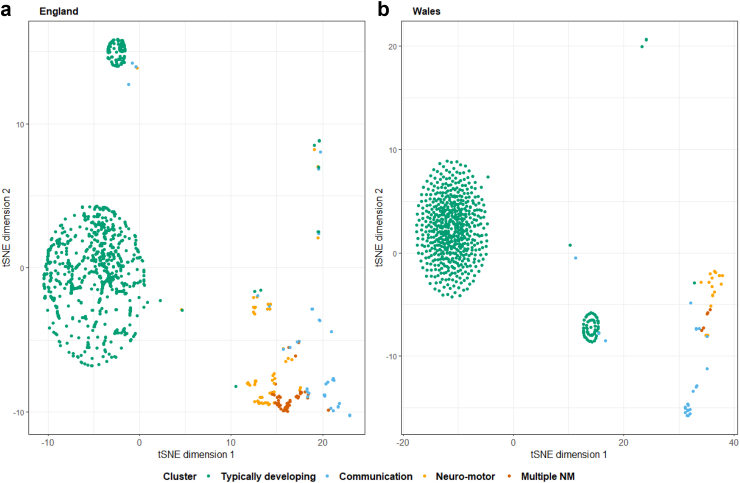
**Two-dimensional representation of thea)England andb)Wales cohorts using t-SNE colour mapped by LCA clusters**.

**Fig. 4:**
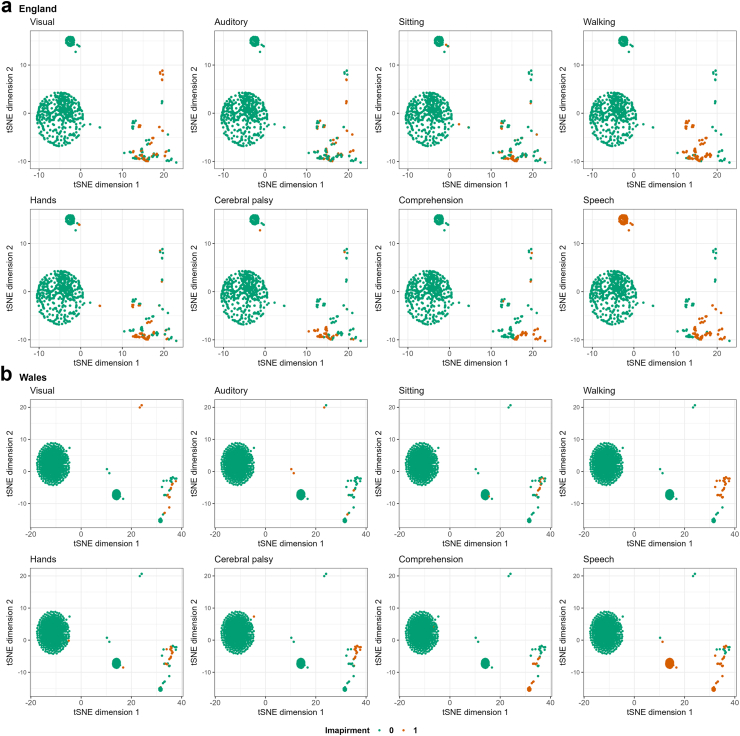
**Two-dimensional representation of thea)England andb)Wales cohorts using t-SNE colour mapped by impairments**.

The t-SNE algorithm was applied to visualise cluster structure (Fig. 3, Fig. 4). TD consisted of two distinct groups: a larger one without impairments and a smaller one characterised exclusively by speech impairments. Children in the TD cluster were twice as likely to have mild rather than severe speech impairments (<10 words: 9% v <5 words: 4%, Figure S4). Other clusters appeared as distinct subgroups, but shared impairments. Both cohorts displayed a similar structure.

### Clinical validation

#### CP type

Figure S5a shows the prevalence of CP type stratified by cluster. There was an absence of CP in TD and COMM. Whilst there was a similar prevalence of CP in NM and MNM (∼80%), a higher proportion of children in the former had hemiplegia or spasticity in two limbs (NM: 55% v MNM: 24%). In contrast, MNM was characterised by a higher presence of spasticity in three or four limbs (NM: 14% v MNM: 44%).

#### BSID-III

Figure S5b–d shows the composite cognitive, language, and motor BSID-III scores for each cluster. TD consistently had the highest mean scores (∼100) across all three assessments, and MNM the lowest (∼60). For cognitive and language assessments, scores were significantly lower for COMM compared with NM. There was no difference in language scores between TD and NM clusters, both of which had a low risk of comprehension and speech impairments. The NM cluster had significantly higher motor scores than the MNM cluster; whilst both are characterised by a similar prevalence of CP, the difference in scores is likely to reflect differences in the type of CP and or the number of motor impairments.

#### HCP assessment of developmental delay

**Fig. 5:**
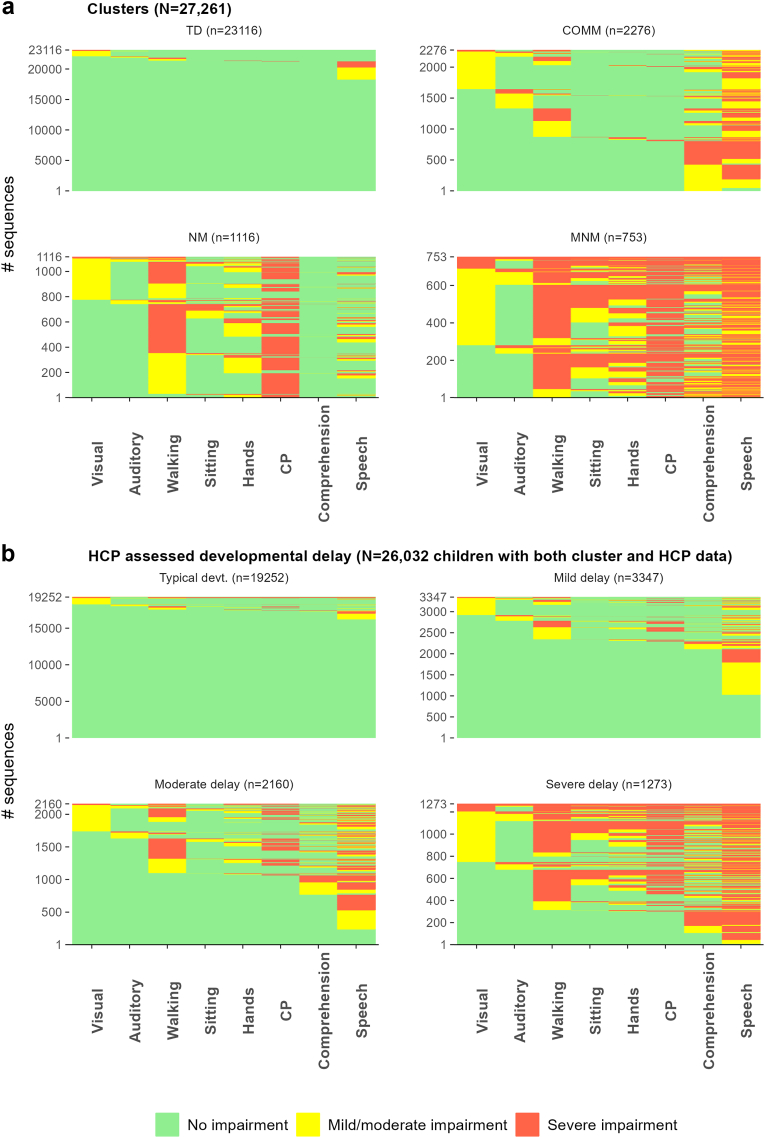
**Individual-level sequences stratified by a) LCA-derived clusters and b) healthcare professional global assessment of developmental delay (England)**. TD = Typically developing, COMM = Communication impairments, NM = Neuro-motor impairments, MNM = Multiple neuro-morbidity. For developmental delay (as diagnosed by a HCP), typically developing: <3 months delay; mild delay: 3–6 months delay; moderate delay: 6–12 months delay; severe delay: >12 months delay. Severity of impairments defined in Table 1. 35% of children assigned to MNM had 5–8 impairments compared with 19% who were assessed as severely delayed.

**Fig. 6:**
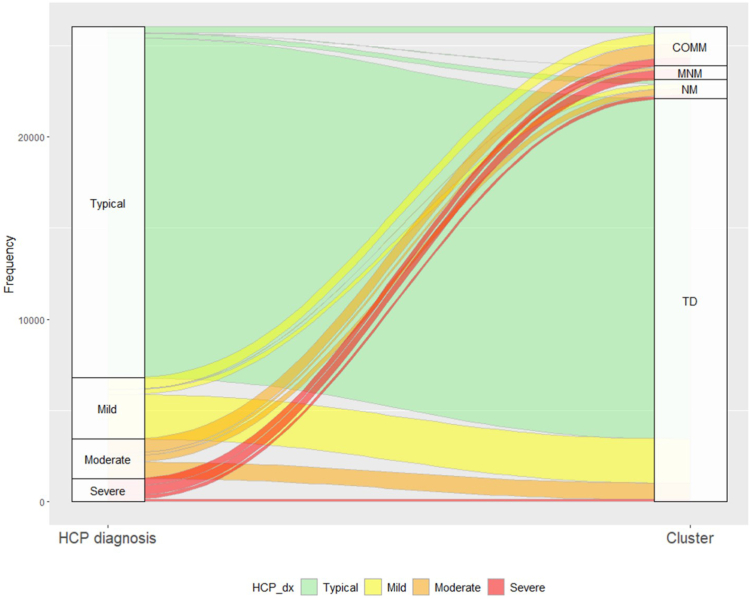
**Alluvial plot to show change in individual assignments between HCP assessment of developmental delay and LCA cluster assignment (England cohort)**. This plot shows that there was low agreement in assignments between both classifications among children with impairments. For example, children from all four developmental delay categories were assigned to the TD cluster. Only 41% with severe developmental delay were assigned to MNM and 10% to TD. N = 26,032 children with both LCA assignment and HCP assessment of developmental delay. Adjusted Rand Index: 0.457.

Fig. 5 visualises individual impairment sequences by cluster and HCP assessment of developmental delay. Although delay was associated with a greater number of impairments, there was high heterogeneity in impairment type and comorbidity patterns across delay categories. All categories included children without impairments, resulting in a lower proportion classified as typically developing (72%) compared to the TD cluster (85%). 30% of children with mild delay and 61% with moderate delay had at least one severe impairment. Twice as many children assigned to MNM had a severe BSID-III motor score compared with those assessed as severely delayed: 61% versus 31%, respectively (Table S3). Overlapping sequences were present across all categories of delay. LCA clusters, however, had mutually exclusive sequences, showing greater internal homogeneity. Consequently, agreement between clusters and HCP assessment was low (Fig. 6, Adjusted Rand Index = 0.457).

### Estimation of cluster-specific antenatal and neonatal features

**Fig. 7:**
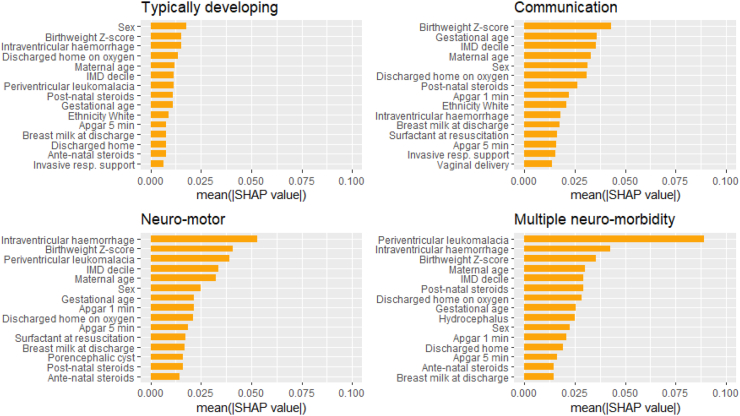
**Global interpretation of the contribution of the top fifteen features to the model predictions of each cluster (England cohort)**. The bar plots show the mean absolute SHAP value of each feature across all observations per cluster in the England cohort. The bars show the impact each feature has on the model predicting each cluster from the RF model. The order of the bars shows the contribution (feature importance) each predictor makes in determining the output of the models (top to bottom = highest to lowest contribution).

Following feature selection using the Boruta algorithm (Figure S7), RF was used to identify the most important features for predicting each cluster. Table S4 shows the distribution of these features by cluster. Fig. 7 shows the mean absolute SHAP value for features for the top fifteen features identified by RF in the England cohort. Figure S8 shows dependence plots for selected features.

Birthweight was the most important neonatal factor associated with clusters; the impact was non-linear with birthweight Z-scores > |2| increasing the risk of all 3 impairment clusters. The impact of gestational age at birth differed by cluster. The proportion of children assigned to TD increased with increasing gestational age (Table S4). 5.1% of children assigned to TD were born at 22–24 weeks compared with 11.9% born at 31 weeks. In contrast, 19.4% in MNM were born at 22–24 weeks compared with 4.2% at the latest gestational age. Gestational age was the second most important feature for COMM but was less predictive than complications of prematurity for NM and MNM. Children born at 22–24 weeks were at increased risk of all three clusters, particularly, COMM. Breast milk at discharge was protective for all clusters.

Cystic periventricular leukomalacia (cPVL) and intraventricular haemorrhage (IVH) were the most important diagnoses for predicting the LCA-derived clusters. The weighted contribution of cPVL was highest for MNM, whereas IVH was a stronger feature for NM. Hydrocephalus predicted MNM, whereas porencephalic cyst was more important for NM. For COMM, perinatal and socio-demographic factors, for example, birthweight, GA, IMD, and sex were more important than comorbidities of preterm birth (Fig. 7).

IMD ranked highly across all clusters, suggesting that socio-economic background is an influential predictor of the clusters. Children born to white mothers had a higher likelihood of TD, whereas children born to Black and Asian mothers had an increased risk of impairments, particularly COMM. Sex had differential effects, with boys less likely than girls to be typically developing and more likely to have communication impairments. Sex differences were less pronounced for NM and MNM. Maternal age was a consistent feature across clusters, with younger (<20 years old) and older (>40 years old) mothers at higher risk of the impairment clusters.

A complete course of antenatal steroids was positively related to TD whereas an absence increased the risk of NM and MNM. Post-natal steroid exposure and the requirement for supplemental oxygen at discharge increased the risk of all three impairment clusters. At 5 min, children with low or intermediate Apgar scores were less likely to be estimated as TD. Children who were discharged to another ward/hospital rather than home were at higher risk of MNM.

### Sensitivity of random forest feature importance to missing feature data

Maternal ethnicity and 5-min Apgar scores had the highest proportion of missingness (10%) (Table S6). There was no missing data for important clinical variables including brain injuries and neonatal morbidities. 13,004 (47.7%) children had missing data for at least one feature (Table S7). Comparison of normalised feature importance scores between complete and imputed data sets showed that variables with the most missing data had relatively low differences in importance scores, suggesting that imputed variables were robust to RF modelling (Figure S9). The largest differences in scores were observed for grade 1–4 IVH and porencephalic cyst, which did not have missing data. This may be due to internal noise in how the RF models are constructed, randomness from running cross-validation or due to very low cases in the TD cluster compared with NM (Table S7). Upon testing on unseen data (Wales), balanced accuracy was higher using data trained on imputed data compared with complete data (0.468 and 0.408, respectively).

## Discussion

LCA applied to routinely collected, whole population, real-world outcomes of preterm infants enabled us to derive four robust and generalisable transdomain clusters of neurodevelopment at two years of age: (1) typically developing (84.8%), (2) communication impairments (8.4%), (3) neuro-motor impairments (4.1%), and (4) multiple neuro-morbidity (2.7%). These replicated with high accuracy in large cohorts from England and Wales. The identified groups corroborate with known phenotypes of neurodevelopmental outcomes in this population.^39^

The clusters had clinical validity against diagnoses and standardised assessments. NM and MNM included children with a high risk of CP; however, the former was characterised by a lower number of and less severe motor impairments. NM children had higher cognitive and language scores using the BSID-III and no comprehension difficulties. MNM not only has the highest number of impairments but also the highest prevalence of severe impairments.

We used the clusters to understand the utility of HCP assessment of global delay, and we demonstrated that agreement between assignments was low. In contrast to the clusters, HCP categories of delay shared overlapping and highly variable impairments. The results suggest that subjective assessments of global developmental delay expressed in months are imprecise in toddlerhood.

Shared and specific maternal and neonatal factors predicted clusters. Cystic PVL and IVH were the most important diagnoses for predicting clusters, with the weighted contribution of cPVL being highest for MNM. Investing in strategies to reduce these forms of preterm brain injury would reduce subsequent neurodisability. The finding that perinatal and sociodemographic factors (birthweight, the degree of prematurity, IMD, and sex) ranked higher than clinical comorbidities for predicting communication impairment supports the notion that environmental/experiential interventions could mitigate some adverse effects of prematurity by leveraging neuroplasticity at a critical period in early life.^39^

Socioeconomic status, operationalised as neighbourhood deprivation, was a consistently important predictor across all clusters, which builds on mounting evidence that the social gradients that shape neurodevelopment in the general population are not obscured by very preterm birth.^40^, ^41^, ^42^ The differential impacts of ethnicity could be due to ethnicity being a proxy of deprivation not captured by IMD, use of English language instruments, or structural racism.^43^

Birthweight z-score was the most important perinatal factor associated with the clusters; the impact was non-linear with birthweight z-scores > |2| increasing the risk of all three impairment clusters. The impact of gestational age at birth differed by cluster, suggesting that foetal growth indexed by weight at birth and gestation length have differential effects on neurodevelopment. As expected, proxies of respiratory morbidity (supplemental oxygen requirement at 36 weeks, requirement for home oxygen) were associated with the impairment clusters. Importantly, we found some modifiable variables were associated with the clusters, including breast milk nutrition at discharge from neonatal care and exposure to corticosteroids (both antenatal and postnatal), which has implications for clinical practice and future research.

Using different domains to our study, the EPIPAGE-2 study applied a similar clustering approach to assess neurodevelopmental outcomes at 5.5 years in 1977 children born <32 weeks' gestation in 2011.^44^ Latent profile analysis identified four clusters: (1) No Deficit (45%), (2) Motor and cognitive deficits (31%), (3) Behavioural and psychosocial deficits (16%), and (4) Multiple deficits (8%). Common to our study, lower gestational age and male sex were associated with the deficit clusters, whereas motor and cognitive deficits were associated with severe neonatal complications. Our study highlights that heterogeneity in neuro-developmental outcomes among very preterm children can be identified before school age using parent-reported functional outcomes.

The study has several strengths. First, use of a contemporary, population-level cohort means that the representativeness of infants with access to modern neonatal intensive care is high. Second, the four clusters were replicated in independent populations with high accuracy. Third, the transdomain approach provides a global overview of neurodevelopment in preterm infants using functional impairments that parents have identified as meaningful and relevant.^45^ This could help reshape prevention and rehabilitation, which are often focused on single domains. Fourth, to our knowledge this is largest study of its kind; the sample size enabled testing of associations of clusters with a large number maternal and neonatal exposures. Fifth, the clusters had clinical validity in that MNM and NM clustered with CP in contrast with TD and COMM which did not cluster with CP. TD had high BSID scores, whereas MNM had low scores. Sixth, whilst recall bias from parental reports is possible, it is unlikely to be significant in our study since parents reported current functioning and needs at the time of assessment. Furthermore, for TRPG-SEND questions on motor, auditory, and visual impairments requiring aids (Table 3), healthcare assessments would have been required to ascertain such need. Overall, these observations increase confidence in the use of parent-reported data as a cost-effective, high-quality data asset for large scale studies.

There are some limitations. First, despite LCA being useful for identifying unobserved heterogeneity, there is no commonly accepted statistical indicator for deciding the number of clusters. Second, whilst parent-reported outcomes showed high agreement with BSID-III scores, assessments were available for only 4–20% of children with a cluster assignment, which could be a source of bias. Third, SES was operationalised by neighbourhood deprivation; it is possible that inclusion of family-level measures may yield different weightings in models. Fourth, with regards to HCP assessment of developmental delay, variability across clinicians is inevitable in routinely collected data. Fifth, severity of the child's impairments may lead to confirmation bias by HCPs, for example, severe delay in one domain may lead to an overall assessment of severe developmental delay.

Future work could include the development of a tool to stratify pre-school children into one of the four clusters which would facilitate targeted early intervention studies. We plan to link cluster allocations with educational records to investigate the impact of pre-school cluster assignment on educational attainment.^20^^,^^46^ Whilst the TRPG-SEND questionnaire made it possible to report significant delays or impairments in the different developmental domains studied at two-years of corrected age, such linkage will help to uncover the longer-term trajectories of preterm-born children. In particular, this research could identify if children in the typically developing cluster persist with good development or experience the onset of mild delay at a later age; children with mild delay are a larger and over-represented group among children with learning difficulties at school age. The perinatal variables that predict cluster membership could be used to inform covariate selection in studies investigating the biological pathways that link preterm birth and its associated co-exposures with atypical brain development.^46^ Whilst our findings generalised to Wales, further studies will be required to ascertain generalisability internationally and for under-represented groups with a two-year assessment record, particularly those from lower socio-economic backgrounds.^37^ Future studies could investigate organisational or neonatal unit-level factors associated with the implementation of protective factors, for example, promoting breast feeding. Finally, a transdisciplinary approach involving neonatologists, parents, data scientists, and policymakers could help advance understanding of outcomes, risk, and protective factors.

## Contributors

All authors read and approved the final version of the manuscript.

Conceptualisation and funding acquisition: JPB, AT, GDB, RMR, SRC, HCW, REM, CB.

Writing – original draft: SH, JPB, CB, AT.

Data curation: SH, CB, JPB, GDB.

Formal analysis and methodology: SH, AT, CB, GDB, JPB.

Supervision: JPB, AT, CB.

Writing – review & editing: all authors.

Investigation: SH, JPB, CB, AT.

Project administration: SH, JPB, CB, AT.

Validation: SH, JPB, CB, AT.

Visualisation: SH, JPB, CB, AT.

Software: SH, AT.

Resources: JPB, AT, GDB, RMR, SRC, HCW, REM, CB.

All authors critically reviewed and commented on the manuscript. All authors take final responsibility for the decision to submit for publication.

## Data sharing statement

NNRD data are available upon reasonable request. Applications to use the data used within this project should be made to the Neonatal Data Analysis Unit, Imperial College London. Requests can be made by researchers to access the NNRD: https://www.imperial.ac.uk/neonatal-data-analysis-unit/neonatal-data-analysis-unit/utilising-the-nnrd/.

## Declaration of interests

AT has involvement in the following grants, which are broadly in the area of digital health, although they did not support the present manuscript: EU Horizon: “EUmetriosis: transforming endometriosis care in Europe via an integrated approach addressing current knowledge, diagnosis, tailored management and patient empowerment” (Universite Catholique de Louvain, Belgium); NIHR: EQUI-RESP-AFRICA (University of Edinburgh); UKRI “AI Centre for Doctoral Training in Biomedical Innovation (AI4BI)” (University of Edinburgh); Wellcome Trust Programme grant: 226944/Z/23/Z (University of Edinburgh); Dunhill Medical Trust PhD studentship: “Preventing rehospitalizations of elderly acute care survivors using longitudinal physical and mental health monitoring with wearable sensors and smartphones” (main PhD supervisor); Exogenous sex steroid hormones and asthma phenotypes: a population-based prospective cohort study using UK-wide primary care databases, (University of Edinburgh); NES Tender, Digital Health and Care Transformation Leaders Programme in Scotland, building on the NHS Digital Academy Leads (University of Edinburgh); UKRI/Versus Arthritis APDP consortia: MR/W002426/1 (University of Cambridge); HEE for the further development of the NHS Digital Academy (renewal award)–collaborative project between Imperial College London, the University of Edinburgh, and HDRUK; Standard Life Grant, topped up by EXPPECT contribution and UoE CMVM funds, PhD studentship on Endometriosis and wearable technology. Supervisors: A. Tsanas, A. Horne, P. Saunders (University of Edinburgh); ESRC: “Beyond the 10,000 steps: Managing less visible aspects of healthy ageing at work” (Business School, University of Edinburgh); BHF: RG/20/10/34966 (University of Edinburgh); HDRUK: CFC0109 (University of Oxford); Wellcome Trust ISSF, 204826/Z/16/Z and 204826/Z/16/Z (University of Oxford); Asthma UK, Asthma: renewal funding bid (University of Edinburgh & QMUL); NHS England commissioning for the development of the NHS Digital Academy. (Imperial College London and the University of Edinburgh, with input from Harvard University); HDRUK core site award (Reg. no: Edin1), Universities of Edinburgh (coordinating), Glasgow, Dundee, Aberdeen, Strathclyde, and St Andrews. AT received consulting fees from Mirador Analytics for statistical risk disclosure and dataset certification. AT received honoraria for talks in the area of digital health (World AI conference) and Cirrus Logic.

SRC reports the following grants: US NIH Grant R01AG054628, U01AG083829, & 1RF1AG073593 (University of Edinburgh); BBSRC & ESRC Grant BB/W008793/1 (University of Edinburgh); Wellcome & Royal Society Grant 221890/Z/20/Z (University of Edinburgh).

CB is supported by National Institute for Health and Care Research (NIHR) via an Advanced Fellowship programme, and holds unpaid leadership roles at the NIHR Health Technology Assessment Prioritisation committee (Deputy Chair) and the British Association of Perinatal Medicine (Honorary Secretary).

JPB holds a MRC UKRI Programme grant: “*Preterm birth as a determinant of neurodevelopment and cognition in children: mechanisms and causal evidence*”, MR/X003434/1, PI: J. Boardman (University of Edinburgh); reports book royalties from Walter Kluwer for Avery and MacDonald's Neonatology Pathophysiology and Management of the Newborn, Eighth edition. Editors: J P Boardman, A M Groves, J Ramasethu. Publisher: Lippincott Williams & Wilkins (LWW). ISBN: 978-1-97-512925-5; support for travel expenses from Perinatal Science International, International Neonatology Association, Witness to House of Lords select committee on preterm birth, and the Joint European Neonatal Societies to attend meetings; participation at Data and Safety Monitoring Committee of the Pregnancy Outcome Prediction Study 2 (POPS2); and holds leadership roles at the NHS England Maternity Neonatal Programme, Member of Clinical Outcomes Group, Scientific Advisory Panel, Action Medical Research, PREMSTEM (Brain injury in the premature born infant: stem cell regeneration research network) scientific advisory board. EU programme.

SH, GDB, RMR, HCW, and REM declare no competing interests.
