# Supplementary material for: Statistical learning to identify and characterise neurodevelopmental outcomes at 2 years in babies born preterm: model development and validation using population-level data from England and Wales
Source: eBioMedicine. 2025 Jun 17;117:105811. doi: 10.1016/j.ebiom.2025.105811 (PMC12212173; doi:10.1016/j.ebiom.2025.105811)
Supplement: Figures and Tables [file mmc1.docx]

**Statistical learning to identify and characterise neurodevelopmental outcomes at 2 years in babies born preterm: model development and validation using population-level data from England and Wales**

Sadia Haider PhD, Athanasios Tsanas DPhil(Oxon), G. David Batty FRSE, Rebecca M Reynolds FMedSci, Heather C Whalley PhD, Simon R Cox PhD, Riccardo E. Marioni PhD, Cheryl Battersby PhD, James P Boardman FMedSci

**Online supplement**

**Table S1: LCA model fit statistics**

AIC = Akaike information criterion; BIC = Bayesian information criterion; aBIC = sample size adjusted BIC; BLRT = bootstrapped likelihood ratio test p-value; ASW=average silhouette width

1. **England cohort (N=27,261 children with complete data on impairments)**

| **Classes** | **AIC** | **BIC** | **aBIC** | **BLRT** | **ASW** |
| --- | --- | --- | --- | --- | --- |
| 1 | 113,635 | 113,700 | 113,675 | – | – |
| 2 | 93,830 | 93,969 | 93,915 | <.001 | 0.785 |
| 3 | 90,112 | 90,326 | 90,243 | <.001 | 0.71 |
| 4 | 88,874 | 89,161 | 89,050 | <.001 | 0.714 |
| 5 | 88,646 | 89,008 | 88,868 | <.001 | 0.772 |
| 6 | 88,553 | 88,988 | 88,820 | <.001 | 0.770 |

1. **Wales (N=975 children with complete data on impairments)**

| **Classes** | **AIC** | **BIC** | **aBIC** | **BLRT** | **ASW** |
| --- | --- | --- | --- | --- | --- |
| 1 | 3,868 | 3,842 | 3,876 | – | – |
| 2 | 3,275 | 3,221 | 3,292 | <.001 | 0.781 |
| 3 | 3,120 | 3,037 | 3,146 | <.001 | 0.726 |
| 4 | 3,125 | 3,014 | 3,160 | <.001 | 0.732 |
| 5 | 3,173 | 3,033 | 3,217 | 0.16 | 0.798 |
| 6 | 3,222 | 3,054 | 3,275 | 0.17 | 0.799 |

# Table S2: Bivariate residuals for the four-class LCA model (England)

|  | Visual | Auditory | Sitting | Walking | Hands | CP | Comprehension |
| --- | --- | --- | --- | --- | --- | --- | --- |
| Auditory | 2.14 |  |  |  |  |  |  |
| Sitting | 0.99 | 1.52 |  |  |  |  |  |
| Walking | 1.32 | 0.04 | 2.57 |  |  |  |  |
| Hands | 1.23 | 0.12 | 1.1 | 0.01 |  |  |  |
| CP | 0.03 | 1.21 | 1.38 | 0.51 | 2.23 |  |  |
| Comprehension | 1.82 | 3.11 | 0.03 | 0.03 | 0.64 | 0.03 |  |
| Speech | 0.01 | 0.03 | 1.12 | 0.01 | 0 | 0.51 | 0.89 |

# Table S3: Distribution of BSID-III scores by clusters and HCP assessment of developmental delay

TD=Typically developing, COMM=Communication impairments, NM-Neuro-motor impairments, MNM=Multiple neuro-morbidity

For developmental delay (as diagnosed by a HCP), typically developing: <3 months delay; mild delay: 3-6 months delay; moderate delay: 6-12 months delay; severe delay: >12 months delay

BSID-III scores categorised as: Normal >=85; mild >=70 & <85; moderate >=55 & <70; severe <55.^1^

| **Clusters** | | | | | | **HCP assessment of developmental delay** | | | | | |
| --- | --- | --- | --- | --- | --- | --- | --- | --- | --- | --- | --- |
|  | **BSID-III language score** | | | |  |  | **BSID-III language score** | | | |  |
| **Cluster** | **Normal** | **Mild** | **Moderate** | **Severe** | **Total** | **Delay** | **Normal** | **Mild** | **Moderate** | **Severe** | **Total** |
| TD | 950 | 168 | 36 | 4 | 1,158 | Typically developing | 749 | 57 | 8 | 0 | 814 |
|  | (82%) | (14.5%) | (3.1%) | (0.4%) | 100 |  | (92%) | (7%) | (1%) | (0%) | 100 |
| COMM | 17 | 39 | 45 | 11 | 112 | Mild delay | 123 | 90 | 14 | 4 | 231 |
|  | (15.2%) | (34.8%) | (40.2%) | (9.8%) | 100 |  | (53.3%) | (39%) | (6.1%) | (1.7%) | 100 |
| NM | 59 | 14 | 2 | 0 | 75 | Moderate delay | 31 | 51 | 35 | 4 | 121 |
|  | (78.7%) | (18.7%) | (2.7%) | (0%) | 100 |  | (25.6%) | (42.2%) | (28.9%) | (3.3%) | 100 |
| MNM | 1 | 3 | 16 | 12 | 32 | Severe delay | 7 | 12 | 39 | 16 | 74 |
|  | (3.1%) | (9.4%) | (50%) | (37.5%) | 100 |  | (9.5%) | (16.2%) | (52.7%) | (21.6%) | 100 |
| Total | 1,027 | 224 | 99 | 27 | 1,377 | Total | 910 | 210 | 96 | 24 | 1,240 |
|  | (74.6%) | (16.3%) | (7.2%) | (2%) | 100 |  | (73.4%) | (16.9%) | (7.7%) | (1.9%) | 100 |
|  | **BSID-III cognitive score** | | | |  |  | **BSID-III cognitive score** | | | |  |
|  | **Normal** | **Mild** | **Moderate** | **Severe** | **Total** |  | **Normal** | **Mild** | **Moderate** | **Severe** | **Total** |
| TD | 4,093 | 314 | 40 | 10 | 4,457 | Typically developing | 3,072 | 60 | 2 | 3 | 3,137 |
|  | (91.8%) | (7.1%) | (0.9%) | (0.2%) | 100 |  | (97.9%) | (1.9%) | (0.1%) | (0.1%) | 100 |
| COMM | 233 | 152 | 124 | 3 | 512 | Mild delay | 669 | 157 | 8 | 5 | 839 |
|  | (45.5%) | (29.7%) | (24.2%) | (0.6%) | 100 |  | (79.7%) | (18.7%) | (1%) | (0.6%) | 100 |
| NM | 159 | 47 | 27 | 1 | 234 | Moderate delay | 317 | 225 | 78 | 0 | 620 |
|  | (68%) | (20.1%) | (11.5%) | (0.4%) | 100 |  | (51.1%) | (36.3%) | (12.6%) | (0%) | 100 |
| MNM | 12 | 29 | 100 | 2 | 143 | Severe delay | 69 | 78 | 186 | 5 | 338 |
|  | (8.4%) | (20.3%) | (69.9%) | (1.4%) | 100 |  | (20.4%) | (23.1%) | (55%) | (1.5%) | 100 |
| Total | 4,497 | 542 | 291 | 16 | 5,346 | Total | 4,127 | 520 | 274 | 13 | 4,934 |
|  | (84.1%) | (10.1%) | (5.4%) | (0.3%) | 100 |  | (83.6%) | (10.5%) | (5.6%) | (0.3%) | 100 |
|  | **BSID-III motor score** | | | |  |  | **BSID-III motor score** | | | |  |
|  | **Normal** | **Mild** | **Moderate** | **Severe** | **Total** |  | **Normal** | **Mild** | **Moderate** | **Severe** | **Total** |
| TD | 865 | 75 | 9 | 0 | 949 | Typically developing | 652 | 26 | 3 | 0 | 681 |
|  | (91.2%) | (7.9%) | (1%) | (0%) | 100 |  | (95.7%) | (3.8%) | (0.4%) | (0%) | 100.0 |
| COMM | 49 | 36 | 20 | 2 | 107 | Mild delay | 140 | 43 | 2 | 0 | 185 |
|  | (45.8%) | (33.6%) | (18.7%) | (1.9%) | 100 |  | (75.7%) | (23.2%) | (1.1%) | (0%) | 100 |
| NM | 19 | 22 | 14 | 1 | 56 | Moderate delay | 36 | 43 | 19 | 0 | 98 |
|  | (33.9%) | (39.3%) | (25%) | (1.8%) | 100 |  | (36.7%) | (43.9%) | (19.4%) | (0%) | 100 |
| MNM | 1 | 3 | 7 | 17 | 28 | Severe delay | 11 | 15 | 19 | 20 | 65 |
|  | (3.6%) | (10.7%) | (25%) | (60.7%) | 100 |  | (16.9%) | (23.1%) | (29.2%) | (30.8%) | 100 |
| Total | 934 | 136 | 50 | 20 | 1,140 | Total | 839 | 127 | 43 | 20 | 1,029 |
|  | (81.9%) | (11.9%) | (4.4%) | (1.8%) | 100 |  | (81.5%) | (12.3%) | (4.2%) | (1.9%) | 100 |

# Table S4: The distribution of features by cluster

**22 and 23 week gestational age categories were collapsed due to small numbers.**

|  | Cluster | | | | |
| --- | --- | --- | --- | --- | --- |
|  | TD | COMM | NM | MNM | Total |
| N | 23,116 (84.8%) | 2,276 (8.3%) | 1,116 (4.1%) | 753 (2.8%) | 27,261 (100.0%) |
| ***Maternal characteristics*** |  |  |  |  |  |
| ***Maternal age (mean (SD))*** | 31 (6.2) | 30.5 (6.3) | 30.3 (6.2) | 29.9 (6.3) | 30.9 (6.2) |
| **Mother's ethnicity** | 15,207 (73.5%) | 1,258 (61.6%) | 801 (79.2%) | 501 (74.2%) | 17,767 (72.7%) |
| White | 2,754 (13.3%) | 353 (17.3%) | 107 (10.6%) | 78 (11.6%) | 3,292 (13.5%) |
| Asian | 1,917 (9.3%) | 337 (16.5%) | 63 (6.2%) | 68 (10.1%) | 2,385 (9.8%) |
| Black |  |  |  |  |  |
| **IMD decile** |  |  |  |  |  |
| 1 - most deprived | 3,608 (16.2%) | 465 (21.2%) | 211 (19.6%) | 156 (21.4%) | 4,440 (16.9%) |
| 2 | 3,051 (13.7%) | 383 (17.4%) | 138 (12.8%) | 117 (16.0%) | 3,689 (14.0%) |
| 3 | 2,695 (12.1%) | 305 (13.9%) | 117 (10.9%) | 90 (12.3%) | 3,207 (12.2%) |
| 4 | 2,375 (10.7%) | 227 (10.3%) | 119 (11.0%) | 70 (9.6%) | 2,791 (10.6%) |
| 5 | 2,160 (9.7%) | 183 (8.3%) | 91 (8.4%) | 71 (9.7%) | 2,505 (9.5%) |
| 6 | 1,915 (8.6%) | 166 (7.6%) | 94 (8.7%) | 59 (8.1%) | 2,234 (8.5%) |
| 7 | 1,825 (8.2%) | 141 (6.4%) | 78 (7.2%) | 53 (7.3%) | 2,097 (8.0%) |
| 8 | 1,687 (7.6%) | 122 (5.6%) | 96 (8.9%) | 48 (6.6%) | 1,953 (7.4%) |
| 9 | 1,602 (7.2%) | 116 (5.3%) | 63 (5.8%) | 36 (4.9%) | 1,817 (6.9%) |
| 10 - least deprived | 1,373 (6.2%) | 90 (4.1%) | 70 (6.5%) | 30 (4.1%) | 1,563 (5.9%) |
| ***Birth factors*** |  |  |  |  |  |
| **Apgar at 1 minute** |  |  |  |  |  |
| Low | 3,561 (17.0%) | 492 (24.2%) | 260 (26.2%) | 213 (32.2%) | 4,526 (18.4%) |
| Intermediate | 7,705 (36.8%) | 843 (41.4%) | 355 (35.8%) | 265 (40.0%) | 9,168 (37.2%) |
| Normal | 9,659 (46.2%) | 702 (34.5%) | 378 (38.1%) | 184 (27.8%) | 10,923 (44.4%) |
| **Apgar at 5 minutes** |  |  |  |  |  |
| Low | 558 (2.7%) | 93 (4.6%) | 48 (4.9%) | 67 (10.1%) | 766 (3.1%) |
| Intermediate | 3,002 (14.4%) | 424 (20.9%) | 233 (23.7%) | 147 (22.2%) | 3,806 (15.6%) |
| Normal | 17,229 (82.9%) | 1,509 (74.5%) | 704 (71.5%) | 448 (67.7%) | 19,890 (81.3%) |
| ***Infant characteristics*** |  |  |  |  |  |
| **Birthweight (z-score) (mean (SD))** | -0.36 (0.88) | -0.54 (0.94) | -0.18 (0.87) | -0.33 (0.85) | -0.37 (0.89) |
| **Gestational age (weeks)** |  |  |  |  |  |
| 22-23 | 292 (1.2%) | 142 (6.2%) | 33 (2.8%) | 53 (7.0%) | 520 (1.9%) |
| 24 | 911 (3.9%) | 265 (11.6%) | 91 (8.2%) | 93 (12.4%) | 1,360 (5.0%) |
| 25 | 1,547 (6.7%) | 263 (11.6%) | 129 (11.6%) | 97 (12.9%) | 2,036 (7.5%) |
| 26 | 2,419 (10.5%) | 287 (12.6%) | 132 (11.8%) | 107 (14.2%) | 2,945 (10.8%) |
| 27 | 3,202 (13.9%) | 316 (13.9%) | 134 (12.0%) | 103 (13.7%) | 3,755 (13.8%) |
| 28 | 4,179 (18.1%) | 343 (15.1%) | 198 (17.7%) | 108 (14.3%) | 4,828 (17.7%) |
| 29 | 4,840 (20.9%) | 312 (13.7%) | 215 (19.3%) | 107 (14.2%) | 5,474 (20.1%) |
| 30 | 2,965 (12.8%) | 200 (8.8%) | 108 (9.7%) | 53 (7.0%) | 3,326 (12.2%) |
| 31 | 2,761 (11.9%) | 148 (6.5%) | 76 (6.8%) | 32 (4.2%) | 3,017 (11.1%) |
| **Sex** |  |  |  |  |  |
| Male | 11,923 (51.6%) | 1,496 (65.7%) | 646 (57.9%) | 470 (62.4%) | 14,535 (53.3%) |
| Female | 11,193 (48.4%) | 780 (34.3%) | 470 (42.1%) | 283 (37.6%) | 12,726 (46.7%) |
| ***Interventions*** |  |  |  |  |  |
| **Antenatal steroids (complete course)** |  |  |  |  |  |
| No | 5,626 (25.6%) | 580 (27.1%) | 375 (35.9%) | 293 (42.2%) | 6,874 (26.6%) |
| Yes | 16,348 (74.4%) | 1,560 (72.9%) | 669 (64.1%) | 401 (57.8%) | 18,978 (73.4%) |
| **Postnatal steroids by 36 wks PMA** |  |  |  |  |  |
| No | 20,839 (91.7%) | 1,708 (76.3%) | 899 (82.3%) | 497 (67.7%) | 23,943 (89.4%) |
| Yes | 1,887 (8.3%) | 532 (23.8%) | 193 (17.7%) | 237 (32.3%) | 2,849 (10.6%) |
| **Surfactant at resuscitation** |  |  |  |  |  |
| No | 8,401 (38.8%) | 523 (24.4%) | 268 (25.3%) | 118 (16.7%) | 9,310 (36.4%) |
| Yes | 13,271 (61.2%) | 1,617 (75.6%) | 791 (74.7%) | 590 (83.3%) | 16,269 (63.6%) |
| **Invasive respiratory support** |  |  |  |  |  |
| No | 7,401 (32.0%) | 417 (18.3%) | 214 (19.2%) | 87 (11.6%) | 8,119 (29.8%) |
| Yes | 15,693 (68.0%) | 1,858 (81.7%) | 900 (80.8%) | 665 (88.4%) | 19,116 (70.2%) |
| ***Neonatal brain injuries*** |  |  |  |  |  |
| **CPVL** |  |  |  |  |  |
| No | 22,942 (99.2%) | 2,234 (98.2%) | 964 (86.4%) | 563 (74.8%) | 26,703 (98.0%) |
| Yes | 174 (0.8%) | 42 (1.8%) | 152 (13.6%) | 190 (25.2%) | 558 (2.0%) |
| **IVH** |  |  |  |  |  |
| No | 19,080 (82.5%) | 1,668 (73.3%) | 682 (61.1%) | 421 (55.9%) | 21,851 (80.2%) |
| Grade 1 or 2 | 3,380 (14.6%) | 469 (20.6%) | 162 (14.5%) | 120 (15.9%) | 4,131 (15.2%) |
| Grade 3 or 4 | 656 (2.8%) | 139 (6.1%) | 272 (24.4%) | 212 (28.2%) | 1,279 (4.7%) |
| **Hydrocephalus** |  |  |  |  |  |
| No | 22,907 (99.1%) | 2,234 (98.2%) | 1,014 (90.9%) | 631 (83.8%) | 26,786 (98.3%) |
| Yes | 209 (0.9%) | 42 (1.8%) | 102 (9.1%) | 122 (16.2%) | 475 (1.7%) |
| ***Discharge*** |  |  |  |  |  |
| **Breast milk at discharge** |  |  |  |  |  |
| No | 15,352 (67.4%) | 1,755 (77.8%) | 817 (75.2%) | 595 (80.8%) | 18,519 (68.9%) |
| Yes | 7,439 (32.6%) | 500 (22.2%) | 269 (24.8%) | 141 (19.2%) | 8,349 (31.1%) |
| **Discharged home** |  |  |  |  |  |
| No | 950 (4.1%) | 218 (9.6%) | 105 (9.4%) | 142 (18.9%) | 1,415 (5.2%) |
| Yes | 22,134 (95.9%) | 2,056 (90.4%) | 1,009 (90.6%) | 610 (81.1%) | 25,809 (94.8%) |
| **Discharged home on oxygen** |  |  |  |  |  |
| No | 17,813 (80.0%) | 1,337 (60.9%) | 753 (70.4%) | 406 (55.8%) | 20,309 (77.3%) |
| Yes | 4,456 (20.0%) | 858 (39.1%) | 316 (29.6%) | 322 (44.2%) | 5,952 (22.7%) |

# Table S5: Frequency and percentage of participants assigned to LCA classes in single and pooled cohorts’ analysis, and adjusted Rand index (ARI) to compare agreement of LCA partitions between each single cohort with the pooled cohorts

TD=Typically developing, COMM=Communication impairments, NM-Neuro-motor impairments, MNM=Multiple neuro-morbidity

There was very high agreement between cluster assignment of individual children when LCA was applied to single or joint cohorts (adjusted Rand index: England v joint=1; Wales v joint =0·99). Only 5 out of 975 children (0·5%) in the Wales cohort changed cluster allocation. This indicates that the classification of impairments in each cohort was consistent and robust to sample size.

|  |  | **England cohort ARI=1** | | | | | **Wales cohort ARI=0.993** | | | | | |
| --- | --- | --- | --- | --- | --- | --- | --- | --- | --- | --- | --- | --- |
| **Partitions of joint cohorts** |  | **TD** | **COMM** | **NM** | **MNM** | **Total** | **TD** | **Comm** | **NM** | **MNM** | **Total** |  |
| **TD** | ***n*** | 23,116 | 0 | 0 | 0 | 23,116 | 827 | 0 | 0 | 0 | 827 |  |
|  | ***%*** | 100 | 0 | 0 | 0 | 100 | 100 | 0 | 0 | 0 | 100 |  |
| **COMM** | ***n*** | 0 | 2,276 | 2 | 0 | 2,278 | 1 | 90 | 0 | 0 | 91 |  |
|  | ***%*** | 0 | 99.9 | 0.1 | 0 | 100 | 1.1 | 98.9 | 0 | 0 | 100 |  |
| **NM** | ***n*** | 0 | 0 | 1,114 | 0 | 1,114 | 0 | 0 | 43 | 0 | 43 |  |
|  | ***%*** | 0 | 0 | 100 | 0 | 100 | 0 | 0 | 100 | 0 | 100 |  |
| **MNM** | ***n*** | 0 | 0 | 0 | 753 | 753 | 0 | 2 | 2 | 10 | 14 |  |
|  | ***%*** | 0 | 0 | 0 | 100 | 100 | 0 | 14.3 | 14.3 | 71.4 | 100 |  |
| **Total** | ***n*** | 23,116 | 2,276 | 1,116 | 753 | 27,261 | 828 | 92 | 45 | 10 | 975 |  |
|  | ***%*** | 84.8 | 8.4 | 4.09 | 2.8 | 100 | 84.9 | 9.4 | 4.6 | 1 | 100 |  |

# Table S6: Prevalence of missing data for features in England cohort for participants with complete records of neurodevelopmental impairments

(N=27,261)

| **Feature** | **N missing** | **% missing** |
| --- | --- | --- |
| **Maternal characteristics** |  |  |
| Maternal ethnicity | 2,832 | 10.4 |
| IMD decile | 965 | 3.5 |
| Mother's age | 146 | 0.5 |
| Maternal smoking | 0 | 0.0 |
| **Pre-existing maternal health conditions** |  |  |
| Mental health | 0 | 0.0 |
| Hypertension | 0 | 0.0 |
| Diabetes | 0 | 0.0 |
| **Maternal obstetric health problems** |  |  |
| Maternal infection | 0 | 0.0 |
| Maternal haemorrhage | 0 | 0.0 |
| Prolonged rupture of membranes | 0 | 0.0 |
| Gestational hypertension | 0 | 0.0 |
| Gestational diabetes | 0 | 0.0 |
| **Infant characteristics** |  |  |
| Extent of prematurity | 0 | 0.0 |
| Birth-weight z-score | 27 | 0.1 |
| Multiple gestation | 3 | 0.0 |
| Sex | 0 | 0.0 |
| SGA | 18 | 0.1 |
| **Birth factors** |  |  |
| Apgar 1 minute | 2,644 | 9.7 |
| Apgar 5 minutes | 2,799 | 10.3 |
| Vaginal delivery | 1,283 | 4.7 |
| **Neonatal comorbidities** |  |  |
| Severe necrotising enterocolitis (NEC) | 0 | 0.0 |
| Treated retinopathy of prematurity (ROP) | 10 | 0.0 |
| Patent ductus arteriosus (PDA) | 10 | 0.0 |
| Late onset sepsis | 0 | 0.0 |
| Late blood growth (any) | 0 | 0.0 |
| **Neonatal brain injuries** |  |  |
| Cystic periventricular leucomalacia (CPVL) | 0 | 0.0 |
| Porencephalic cyst | 0 | 0.0 |
| Hydrocephalus | 0 | 0.0 |
| Intraventricular haemorrhage | 0 | 0.0 |
| **Interventions** |  |  |
| Ante-natal steroids (complete course) | 1,409 | 5.2 |
| Surfactant at resuscitation | 1,682 | 6.2 |
| Invasive respiratory support | 26 | 0.1 |
| Non-invasive respiratory support | 26 | 0.1 |
| Post-natal steroids | 469 | 1.7 |
| Antibiotics by 36 weeks' PMA | 468 | 1.7 |
| **Discharge** |  |  |
| Discharged on oxygen | 1,000 | 3.7 |
| Discharged on breast milk | 393 | 1.4 |
| Discharge destination | 37 | 0.1 |
| **Bayley Scales of Toddler & Infant Development (Third edition) composite scores** | | |
| Language | 25,884 | 94.9 |
| Cognitive | 21,915 | 80.4 |
| Motor | 26,121 | 95.8 |

# Table S7: Prevalence of features stratified by participants with missing and complete data on covariates

|  | Sample with: | |  |
| --- | --- | --- | --- |
|  | >=1 missing feature | complete features | Total |
| **N** | 13,004 (47.7%) | 14,257 (52.3%) | 27,261 (100.0%) |
| ***Maternal characteristics*** |  |  |  |
| ***Maternal age (mean (SD))*** | 30.8 (6.3) | 30.9 (6.2) | 30.9 (6.2) |
| **Mother's ethnicity** |  |  |  |
| White | 7,533 (74.1%) | 10,234 (71.8%) | 17,767 (72.7%) |
| Asian | 1,260 (12.4%) | 2,032 (14.3%) | 3,292 (13.5%) |
| Black | 963 (9.5%) | 1,422 (10.0%) | 2,385 (9.8%) |
| **Maternal smoking** |  |  |  |
| No | 11,085 (85.2%) | 12,020 (84.3%) | 23,105 (84.8%) |
| Yes | 1,919 (14.8%) | 2,237 (15.7%) | 4,156 (15.2%) |
| **IMD decile** |  |  |  |
| 1 - most deprived | 2,204 (18.3%) | 2,236 (15.7%) | 4,440 (16.9%) |
| 2 | 1,751 (14.5%) | 1,938 (13.6%) | 3,689 (14.0%) |
| 3 | 1,422 (11.8%) | 1,785 (12.5%) | 3,207 (12.2%) |
| 4 | 1,250 (10.4%) | 1,541 (10.8%) | 2,791 (10.6%) |
| 5 | 1,121 (9.3%) | 1,384 (9.7%) | 2,505 (9.5%) |
| 6 | 993 (8.2%) | 1,241 (8.7%) | 2,234 (8.5%) |
| 7 | 909 (7.6%) | 1,188 (8.3%) | 2,097 (8.0%) |
| 8 | 857 (7.1%) | 1,096 (7.7%) | 1,953 (7.4%) |
| 9 | 862 (7.2%) | 955 (6.7%) | 1,817 (6.9%) |
| 10 -least deprived | 670 (5.6%) | 893 (6.3%) | 1,563 (5.9%) |
| ***Maternal obstetric health problems*** |  |  |  |
| **Gestational hypertension** |  |  |  |
| No | 11,746 (90.3%) | 12,382 (86.8%) | 24,128 (88.5%) |
| Yes | 1,258 (9.7%) | 1,875 (13.2%) | 3,133 (11.5%) |
| **Maternal infection** |  |  |  |
| No | 7,519 (57.8%) | 6,448 (45.2%) | 13,967 (51.2%) |
| Yes | 5,485 (42.2%) | 7,809 (54.8%) | 13,294 (48.8%) |
| **Prolonged rupture of membrane** |  |  |  |
| No | 10,494 (80.7%) | 10,608 (74.4%) | 21,102 (77.4%) |
| Yes | 2,510 (19.3%) | 3,649 (25.6%) | 6,159 (22.6%) |
| ***Birth factors*** |  |  |  |
| **Mode of delivery** |  |  |  |
| C-section | 4,878 (41.6%) | 5,955 (41.8%) | 10,833 (41.7%) |
| Vaginal | 6,843 (58.4%) | 8,302 (58.2%) | 15,145 (58.3%) |
| **Apgar at 1 minute** |  |  |  |
| Low | 1,966 (19.0%) | 2,560 (18.0%) | 4,526 (18.4%) |
| Intermediate | 3,803 (36.7%) | 5,365 (37.6%) | 9,168 (37.2%) |
| Normal | 4,591 (44.3%) | 6,332 (44.4%) | 10,923 (44.4%) |
| **Apgar at 5 minutes** |  |  |  |
| Low | 344 (3.4%) | 422 (3.0%) | 766 (3.1%) |
| Intermediate | 1,605 (15.7%) | 2,201 (15.4%) | 3,806 (15.6%) |
| Normal | 8,256 (80.9%) | 11,634 (81.6%) | 19,890 (81.3%) |
| ***Infant characteristics*** |  |  |  |
| **Birthweight (z-score) (mean (SD))** | -0.35 (0.89) | -0.38 (0.89) | -0.37 (0.89) |
| **Degree of prematurity (gestational age (weeks))** |  |  |  |
| Very preterm (28-32 weeks) | 7,843 (60.3%) | 8,802 (61.7%) | 16,645 (61.1%) |
| Extremely preterm (25-27 weeks) | 4,307 (33.1%) | 4,429 (31.1%) | 8,736 (32.0%) |
| Limits of viability (22-24 weeks) | 854 (6.6%) | 1,026 (7.2%) | 1,880 (6.9%) |
| **Sex assigned at birth** |  |  |  |
| Male | 6,968 (53.6%) | 7,567 (53.1%) | 14,535 (53.3%) |
| Female | 6,036 (46.4%) | 6,690 (46.9%) | 12,726 (46.7%) |
| **Small for gestational age** |  |  |  |
| No | 11,019 (84.9%) | 12,010 (84.2%) | 23,029 (84.5%) |
| Yes | 1,967 (15.1%) | 2,247 (15.8%) | 4,214 (15.5%) |
| **Multiple birth** |  |  |  |
| Singleton | 9,342 (71.9%) | 10,535 (73.9%) | 19,877 (72.9%) |
| Multiple | 3,659 (28.1%) | 3,722 (26.1%) | 7,381 (27.1%) |
| ***Interventions*** |  |  |  |
| **Antenatal steroids (complete course)** |  |  |  |
| No | 3,110 (26.8%) | 3,764 (26.4%) | 6,874 (26.6%) |
| Yes | 8,485 (73.2%) | 10,493 (73.6%) | 18,978 (73.4%) |
| **Intrapartum antibiotics** |  |  |  |
| No | 3,823 (70.7%) | 9,679 (67.9%) | 13,502 (68.6%) |
| Yes | 1,588 (29.3%) | 4,578 (32.1%) | 6,166 (31.4%) |
| **Surfactant at resuscitation** |  |  |  |
| No | 3,821 (33.7%) | 5,489 (38.5%) | 9,310 (36.4%) |
| Yes | 7,501 (66.3%) | 8,768 (61.5%) | 16,269 (63.6%) |
| **Antibiotics by 36 wks PMA** |  |  |  |
| No | 318 (2.5%) | 267 (1.9%) | 585 (2.2%) |
| Yes | 12,218 (97.5%) | 13,990 (98.1%) | 26,208 (97.8%) |
| **Postnatal steroids by 36 wks PMA** |  |  |  |
| No | 11,184 (89.2%) | 12,759 (89.5%) | 23,943 (89.4%) |
| Yes | 1,351 (10.8%) | 1,498 (10.5%) | 2,849 (10.6%) |
| **Invasive respiratory support** |  |  |  |
| No | 4,071 (31.4%) | 4,048 (28.4%) | 8,119 (29.8%) |
| Yes | 8,907 (68.6%) | 10,209 (71.6%) | 19,116 (70.2%) |
| **Non-invasive respiratory support** |  |  |  |
| No | 474 (3.7%) | 497 (3.5%) | 971 (3.6%) |
| Yes | 12,504 (96.3%) | 13,760 (96.5%) | 26,264 (96.4%) |
| ***Neonatal comorbidities*** |  |  |  |
| **Severe NEC** |  |  |  |
| No | 12,655 (97.3%) | 13,898 (97.5%) | 26,553 (97.4%) |
| Yes | 349 (2.7%) | 359 (2.5%) | 708 (2.6%) |
| **Treated ROP** |  |  |  |
| No | 12,394 (95.4%) | 13,540 (95.0%) | 25,934 (95.2%) |
| Yes | 600 (4.6%) | 717 (5.0%) | 1,317 (4.8%) |
| **Late onset sepsis** |  |  |  |
| No | 12,511 (96.2%) | 13,641 (95.7%) | 26,152 (95.9%) |
| Yes | 493 (3.8%) | 616 (4.3%) | 1,109 (4.1%) |
| **Late blood infection (any growth)** |  |  |  |
| No | 11,429 (87.9%) | 12,173 (85.4%) | 23,602 (86.6%) |
| Yes | 1,575 (12.1%) | 2,084 (14.6%) | 3,659 (13.4%) |
| **PDA** |  |  |  |
| No | 12,620 (97.1%) | 13,890 (97.4%) | 26,510 (97.3%) |
| Yes | 374 (2.9%) | 367 (2.6%) | 741 (2.7%) |
| ***Neonatal brain injuries*** |  |  |  |
| **CPVL** |  |  |  |
| No | 12,750 (98.0%) | 13,953 (97.9%) | 26,703 (98.0%) |
| Yes | 254 (2.0%) | 304 (2.1%) | 558 (2.0%) |
| **IVH** |  |  |  |
| No | 10,550 (81.1%) | 11,301 (79.3%) | 21,851 (80.2%) |
| Grade 1 or 2 | 1,843 (14.2%) | 2,288 (16.0%) | 4,131 (15.2%) |
| Grade 3 or 4 | 611 (4.7%) | 668 (4.7%) | 1,279 (4.7%) |
| **Porencephalic cyst** |  |  |  |
| No | 12,812 (98.5%) | 13,978 (98.0%) | 26,790 (98.3%) |
| Yes | 192 (1.5%) | 279 (2.0%) | 471 (1.7%) |
| **Hydrocephalus** |  |  |  |
| No | 12,779 (98.3%) | 14,007 (98.2%) | 26,786 (98.3%) |
| Yes | 225 (1.7%) | 250 (1.8%) | 475 (1.7%) |
| ***Discharge*** |  |  |  |
| **Breast milk at discharge** |  |  |  |
| No | 8,843 (70.1%) | 9,676 (67.9%) | 18,519 (68.9%) |
| Yes | 3,768 (29.9%) | 4,581 (32.1%) | 8,349 (31.1%) |
| **Discharged home** |  |  |  |
| No | 847 (6.5%) | 568 (4.0%) | 1,415 (5.2%) |
| Yes | 12,120 (93.5%) | 13,689 (96.0%) | 25,809 (94.8%) |
| **Discharged home on oxygen** |  |  |  |
| No | 9,246 (77.0%) | 11,063 (77.6%) | 20,309 (77.3%) |
| Yes | 2,758 (23.0%) | 3,194 (22.4%) | 5,952 (22.7%) |
| ***Cluster*** |  |  |  |
| Typically developing | 10,971 (84.4%) | 12,145 (85.2%) | 23,116 (84.8%) |
| Communication | 1,091 (8.4%) | 1,185 (8.3%) | 2,276 (8.3%) |
| Neuromotor | 566 (4.4%) | 550 (3.9%) | 1,116 (4.1%) |
| Multiple neuro-morbid | 376 (2.9%) | 377 (2.6%) | 753 (2.8%) |

# Table S8: Comparison of RF accuracy following different class rebalancing strategies for the prediction of cluster ante- and peri-natal features

Data trained on England cohort and tested on Wales cohort.

For estimating the features of each cluster using RF, balanced accuracy was low for each strategy when tested on the Wales cohort, possibly due to the highly imbalanced clusters and shared features across clusters. Training on England data using imbalanced classes yielded the highest balanced accuracy score (0.47), hence, we opted to present the results from RF models using the imbalanced classes. Furthermore, the cluster sizes reflected the distribution expected clinically in the real-world and there were a relatively large number of observations in the minority class (~800).

|  | **Test on Wales** | |
| --- | --- | --- |
|  | **Mean accuracy** | **Balanced accuracy** |
| 1. Training on England cohort using imbalanced classes | 0.847 | 0.470 |
| 2. Under-sample (all class sizes=size of minority) | 0.531 | 0.373 |
| 4. SMOTE (over-sample to 50% of majority) | 0.796 | 0.347 |
| 5. Weighted classes | 0.850 | 0.292 |

# Figure S1: Participant flow for included children born before 32 completed weeks gestation between 1st January 2007 and 31st December 2019 and admitted to neonatal units in England and Wales with complete records on parent-reported neuro-developmental impairments

88,473/100,631 (88%) babies born before 32 weeks’ gestation were admitted to neonatal units and survived to discharge between 2007-2019. 374 died post-discharge but before 2 years. 33,533/88,099 (38%) had a neurodevelopmental record at 2 years corrected gestational age. Among these, 32,125 children had at least one non-missing record on the eight impairments. 27,261/31,042 (88%) children in the England cohort and 975/1098 (89%) in the Wales cohort with complete information on all impairments were included in the analysis.


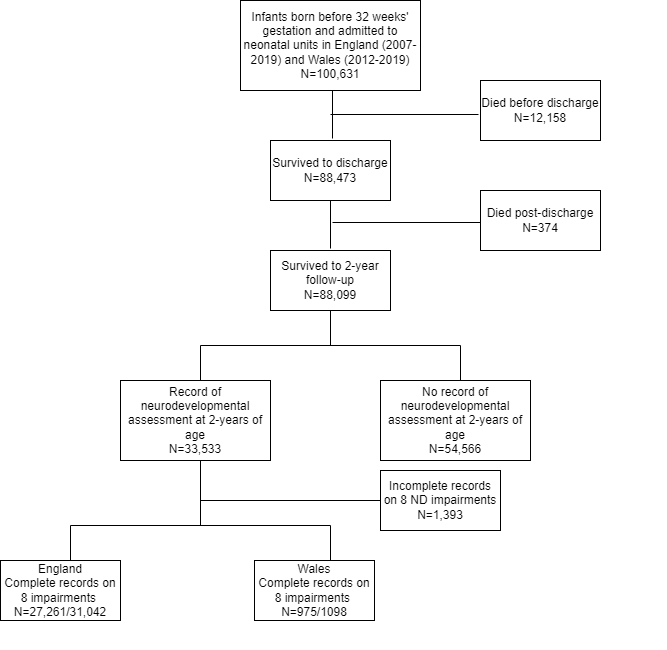


# Figure S2: Elbow plot of AIC, BIC, sample-size adjusted BIC and average silhouette width

1. **England information criteria**


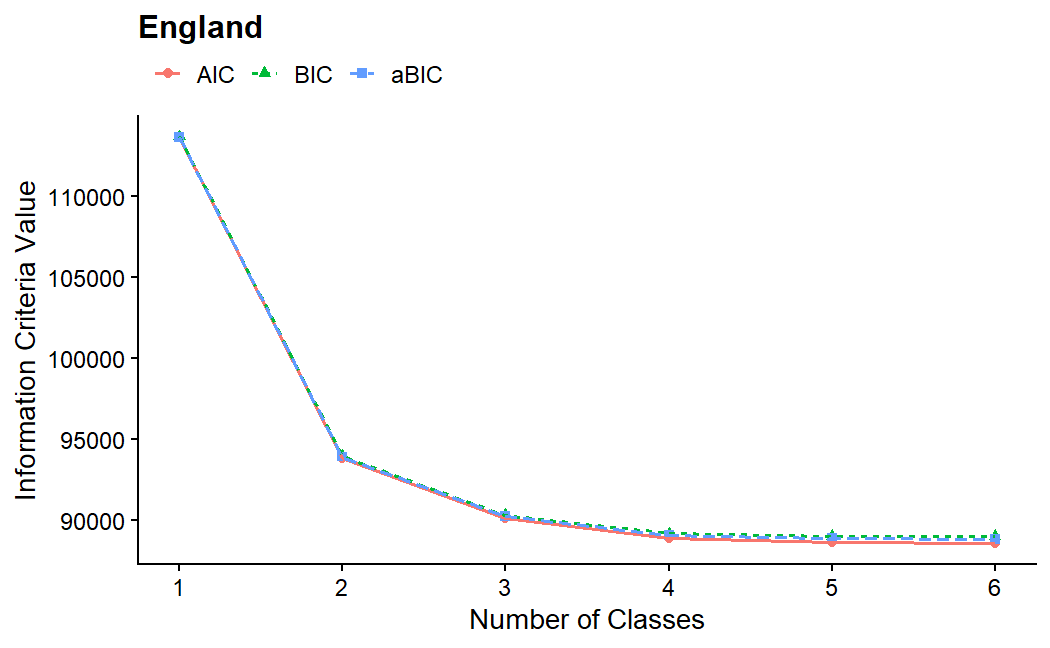


1. **Wales information criteria**

**
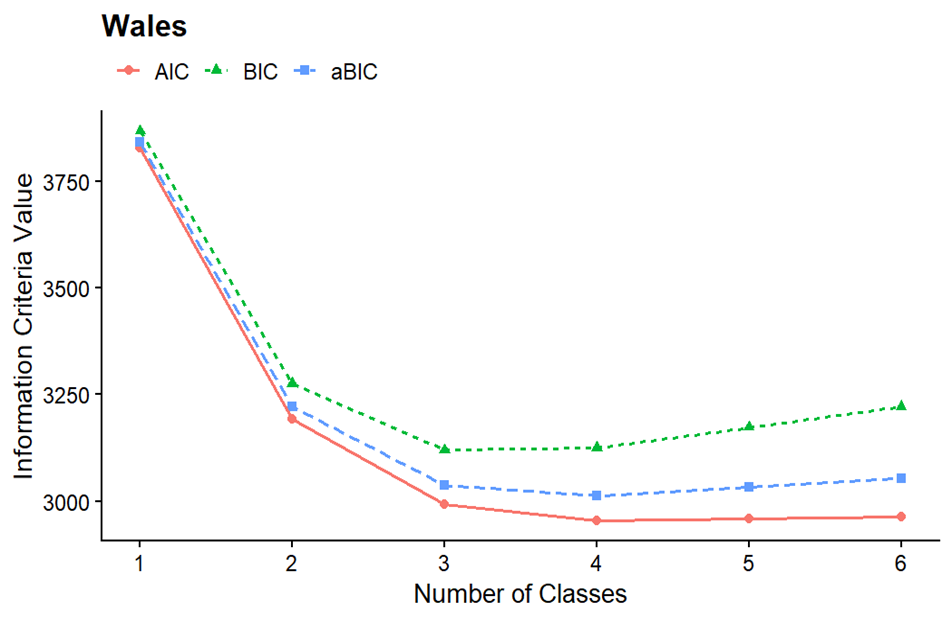
**

1. **England average silhouette width for 4 clusters**

TD=Typically developing, COMM=Communication impairments, NM-Neuro-motor impairments, MNM=Multiple neuro-morbidity

**
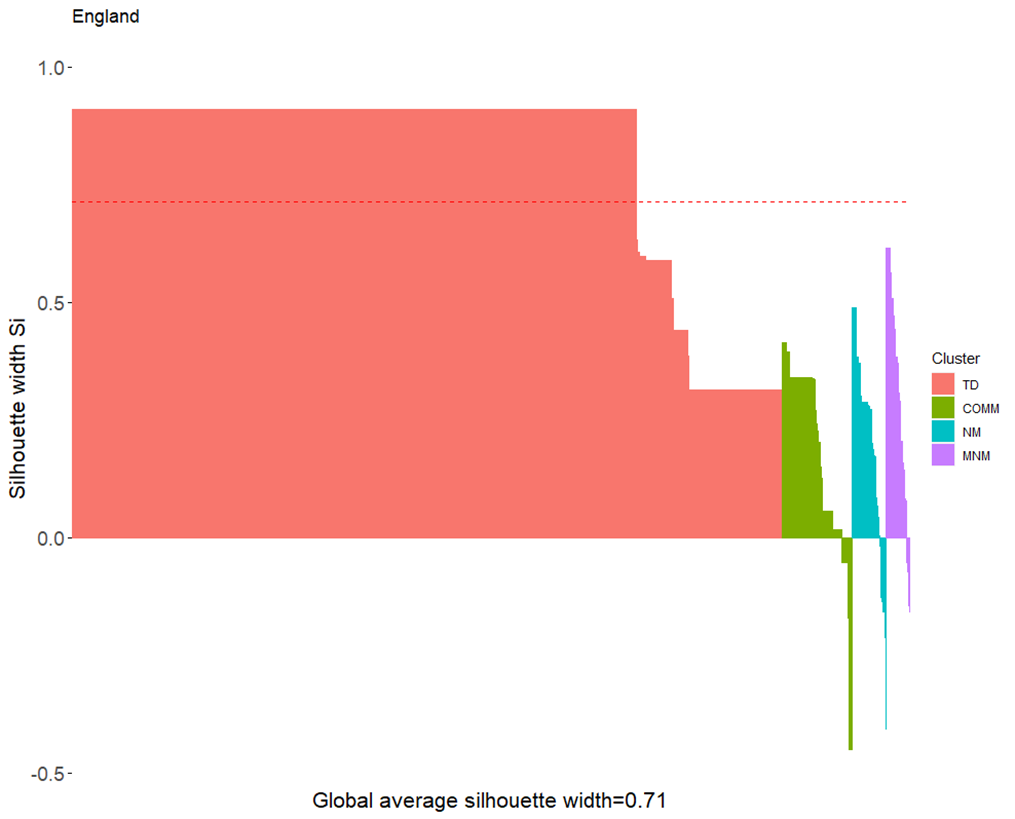
**

1. **Wales average silhouette width for 4 clusters**

**
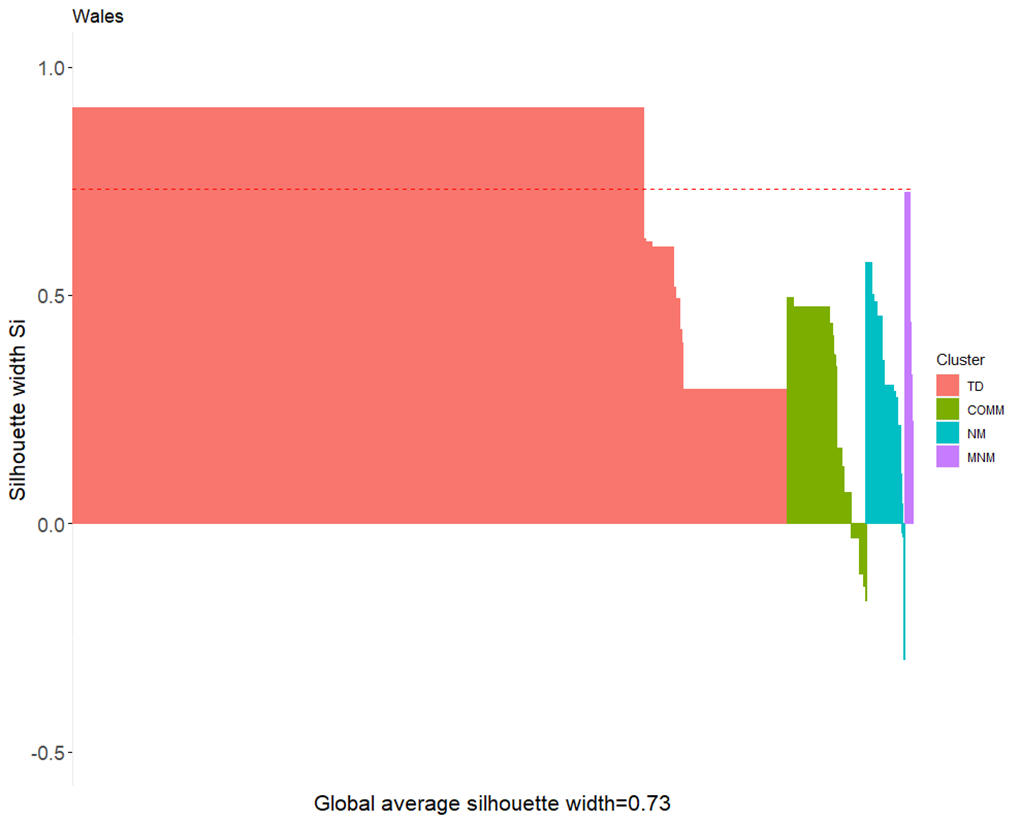
**

# Figure S3: Evolution of clusters with additional classes

Each plot shows the prevalence of each impairment within each cluster in 1 to 6-class models.

A new cluster was identified in the four-cluster model compared to the three-cluster one. These four remained in subsequent models. The additional cluster in the five-cluster model ‘splintered’ from larger groups in the four-cluster model and was not deemed clinically meaningful.

1. **England**


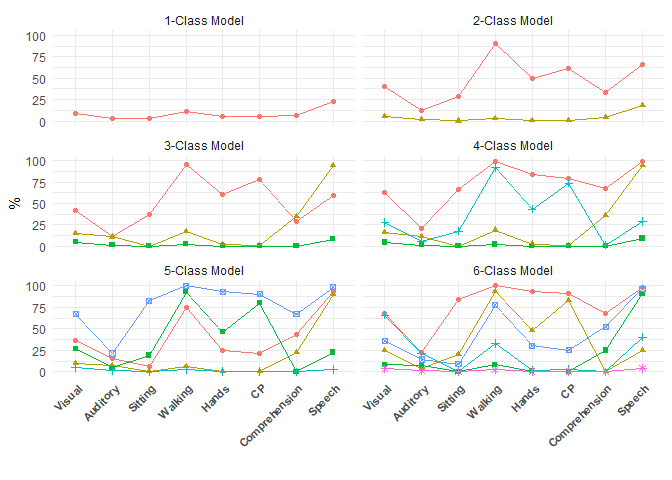


1. **Wales**

**
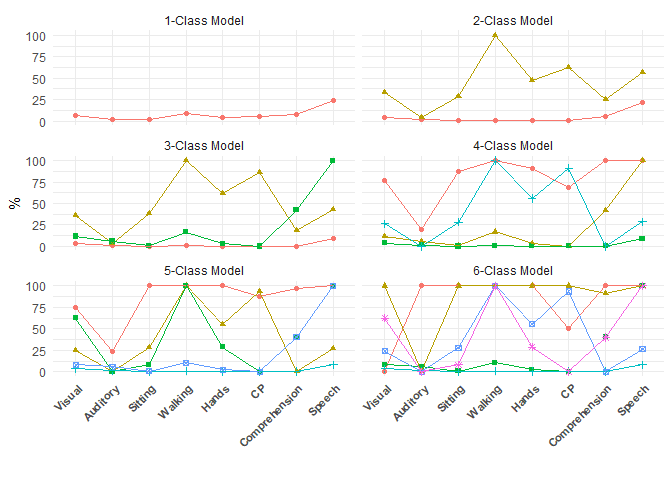
**

# **Figure S4: Severity of speech impairments by cluster**

1. **England**

**
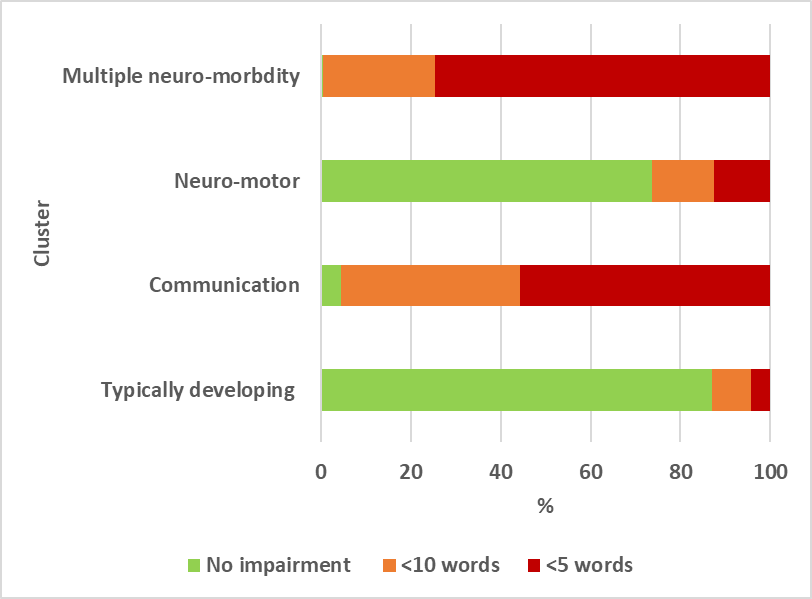
**

1. **Wales**

**
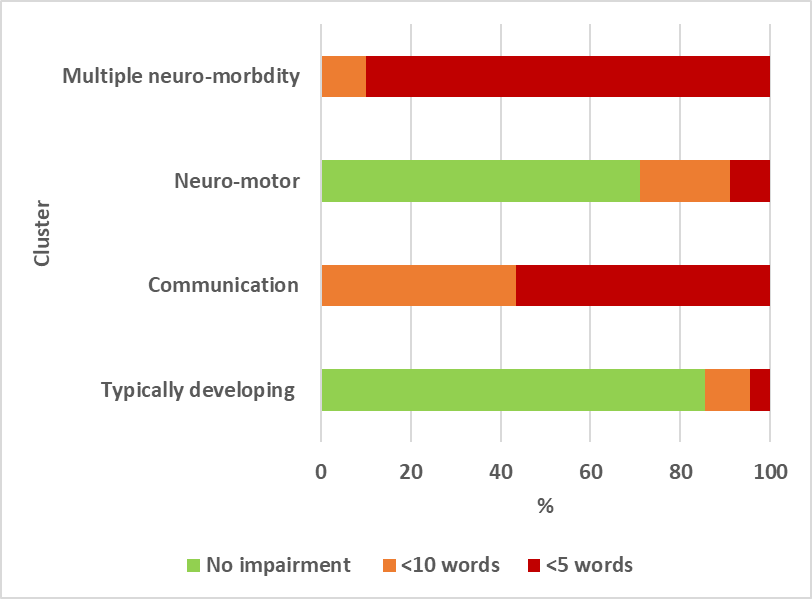
**

# Figure S5: The prevalence of cerebral palsy type and mean BSID-III composite language, cognitive, and motor scores by cluster (England cohort)

(Language score: N=1377/27261; Cognitive score: N=5346/27261; Motor score: N=1140/27261)

TD=Typically developing, COMM=Communication impairments, NM-Neuro-motor impairments, MNM=Multiple neuro-morbidity

Sp. Bil=spastic bilaterality; L/R-left/right limb

1. **Prevalence of cerebral palsy type**


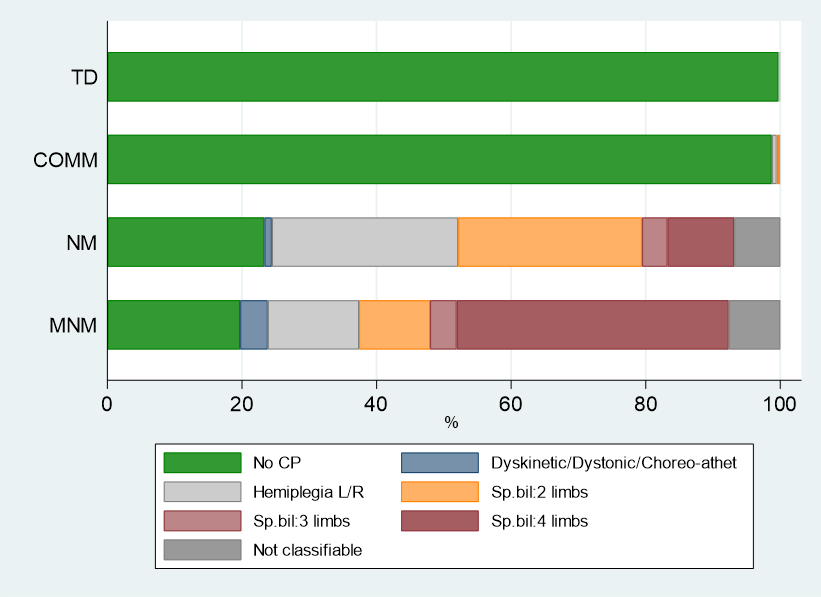


1. **Mean (95% CI) BSID-III composite language score**

1. **Mean (95% CI) BSID-III composite cognitive score**

1. **Mean (95% CI) BSID-III composite motor score**

# Figure S6: Confusion matrices for the prediction of class labels in test data using random forest classification

10-fold cross validation with 10 repetitions was carried out on the training data.

a)       The model was trained on England and tested on Wales. Overall balanced accuracy: validation =0.999; test=0.925

b)      The model was trained on Wales and tested on England. Overall balanced accuracy: validation =0.960; test=0.923


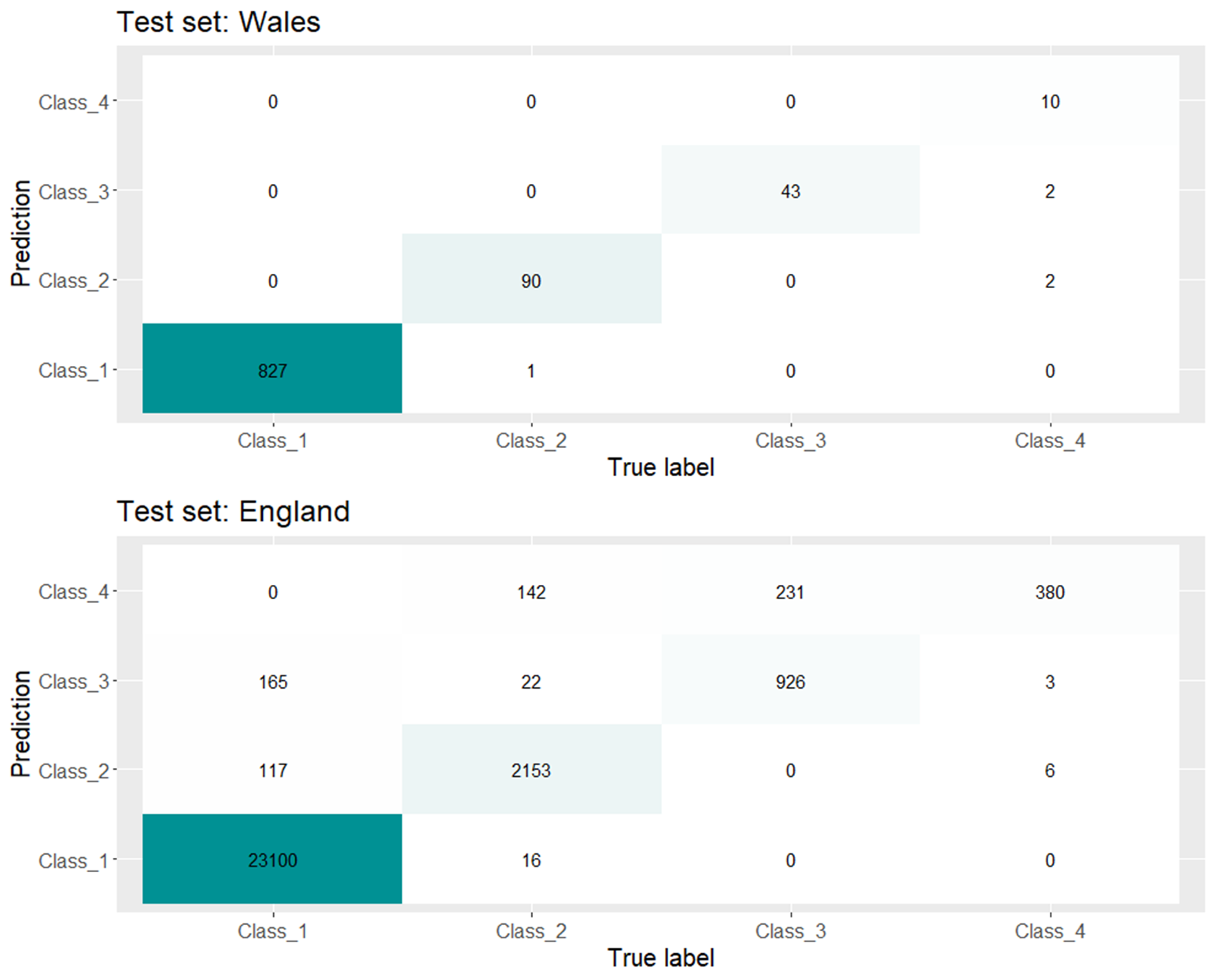


# Figure S7: Features selected by Boruta algorithm (England cohort)

The green, yellow, and red boxplots represent the z-scores of the confirmed, tentative, and rejected features respectively.

The Boruta algorithm selected 36/43 features as important predictors of clusters. 7 maternal features were rejected, namely, diabetes (pre-exiting and gestational), pre-existing hypertension, mental health problems, pregnancy-related haemorrhage, and Mixed and Other ethnicity.

An out-of-bag imputation error of 0.00407 was achieved.


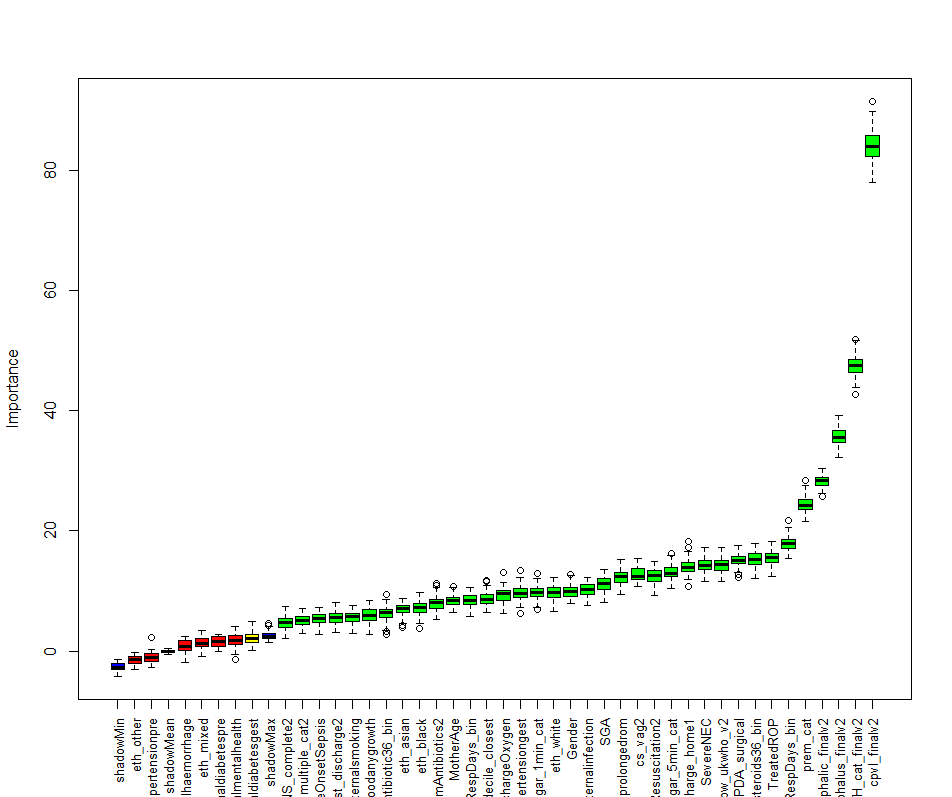


# Figure S8: SHAP dependence plots showing the impact of a change in each feature's value on the prediction of each cluster (England cohort)

1. **Cystic periventricular leukomalacia**

**
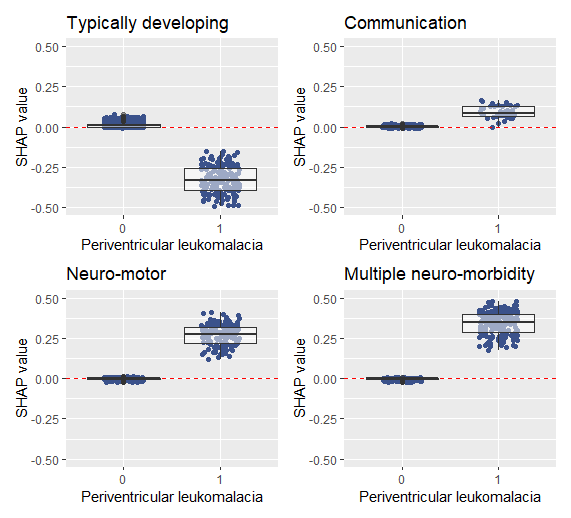
**

1. **Intraventricular haemorrhage (IVH)**

**
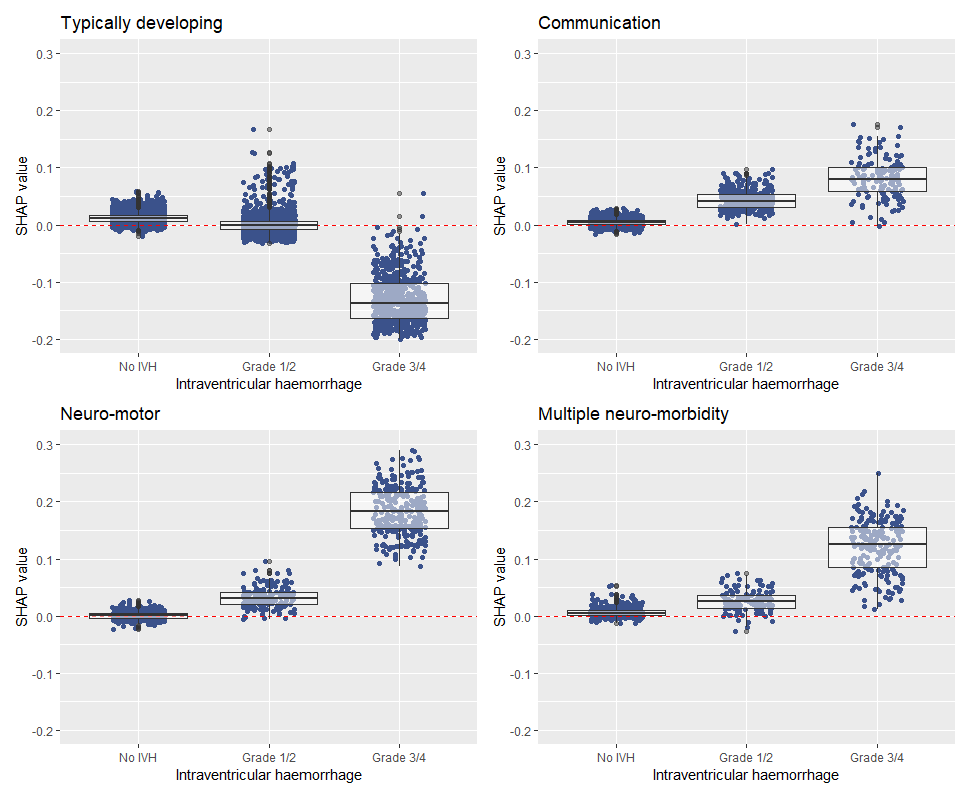
**

1. **Sex**

**
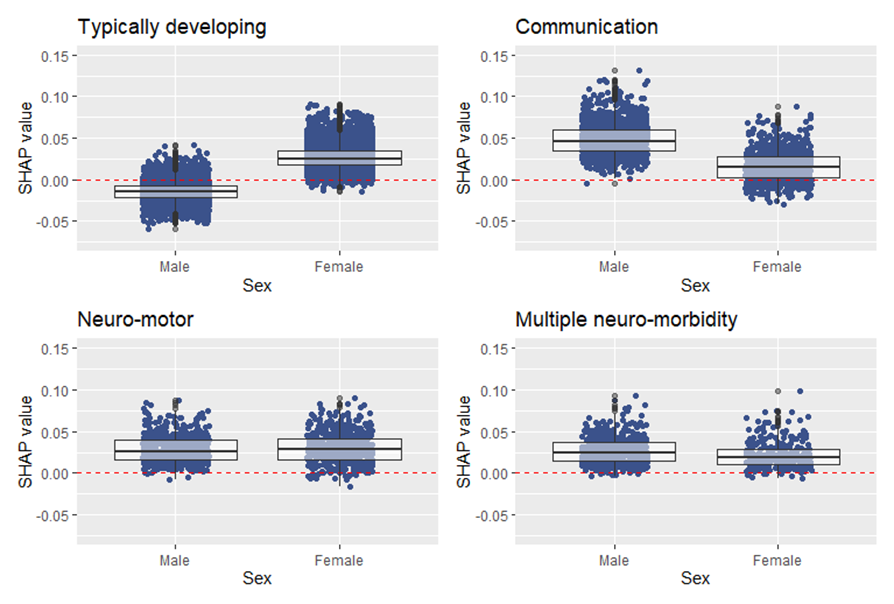
**

1. **Gestational age (weeks)**

**
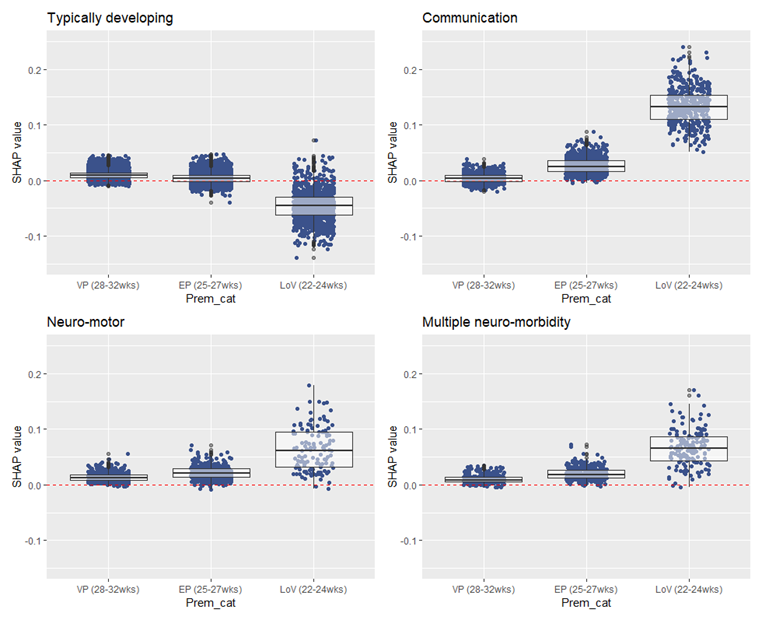
**

1. **Birthweight z-score coloured by gestational age**


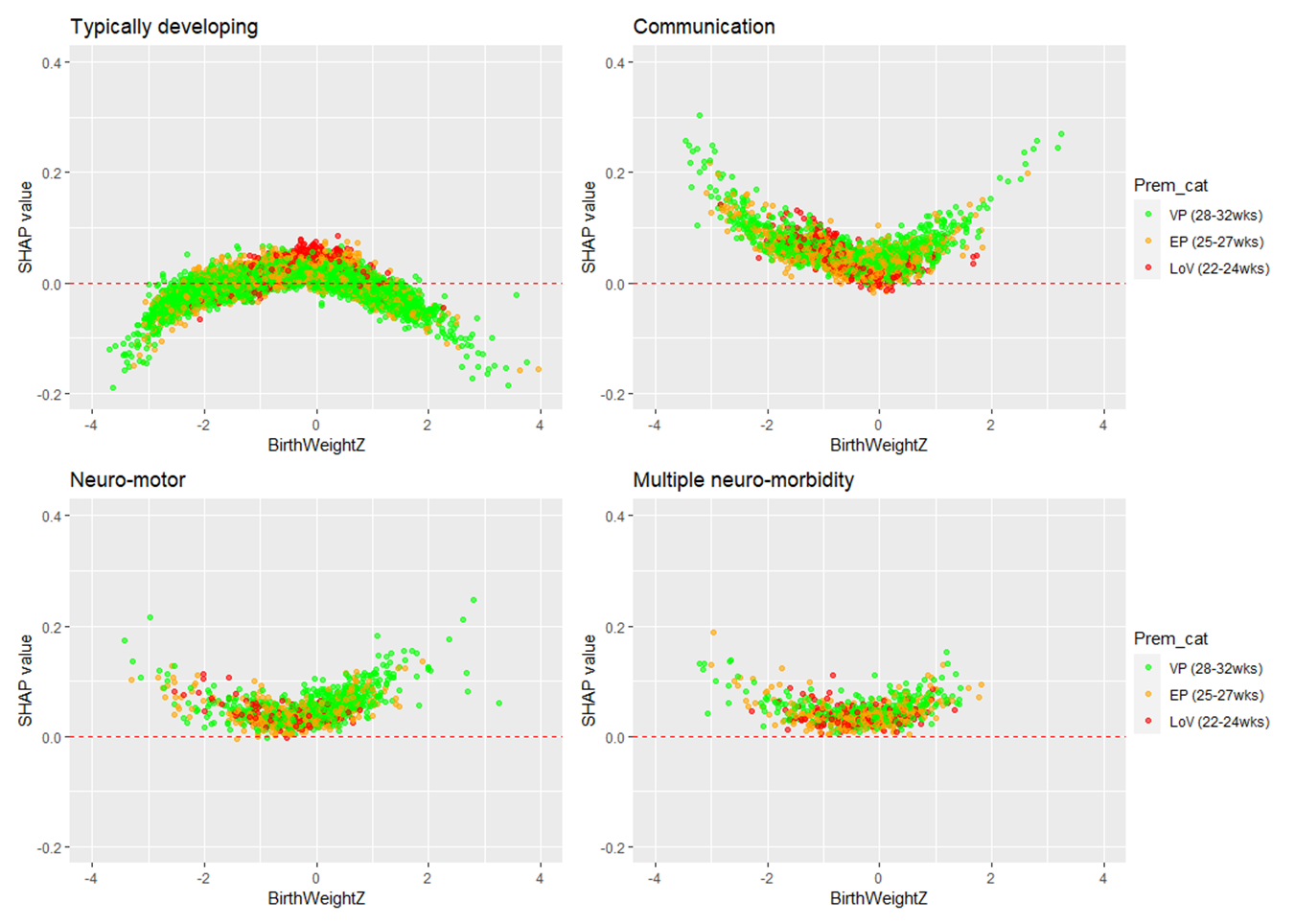


1. **IMD decile (1=most deprived, 10=least deprived)**


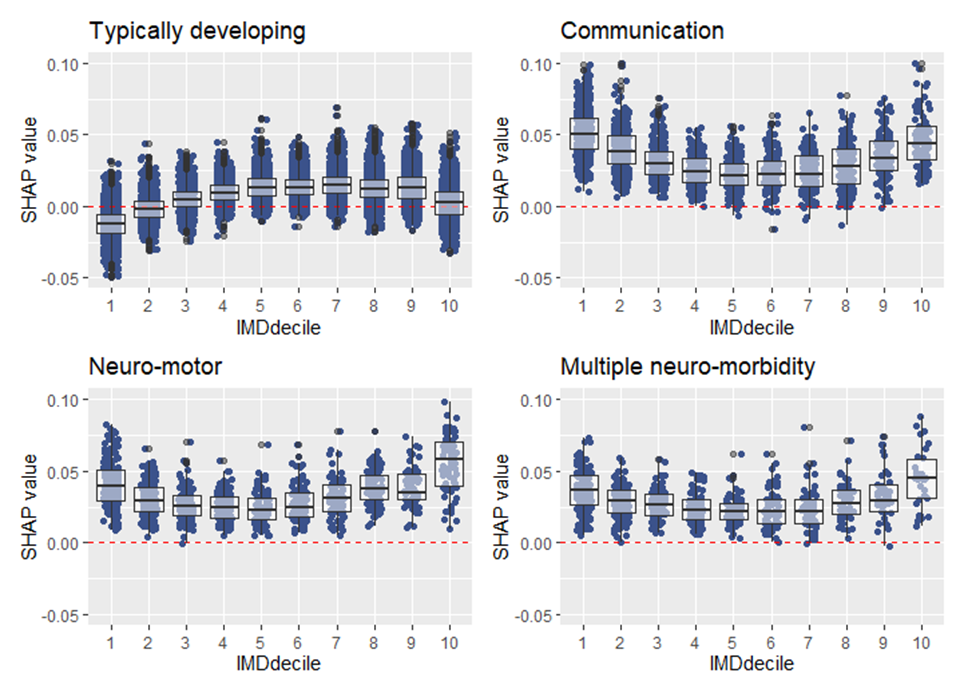


1. **Ethnicity**


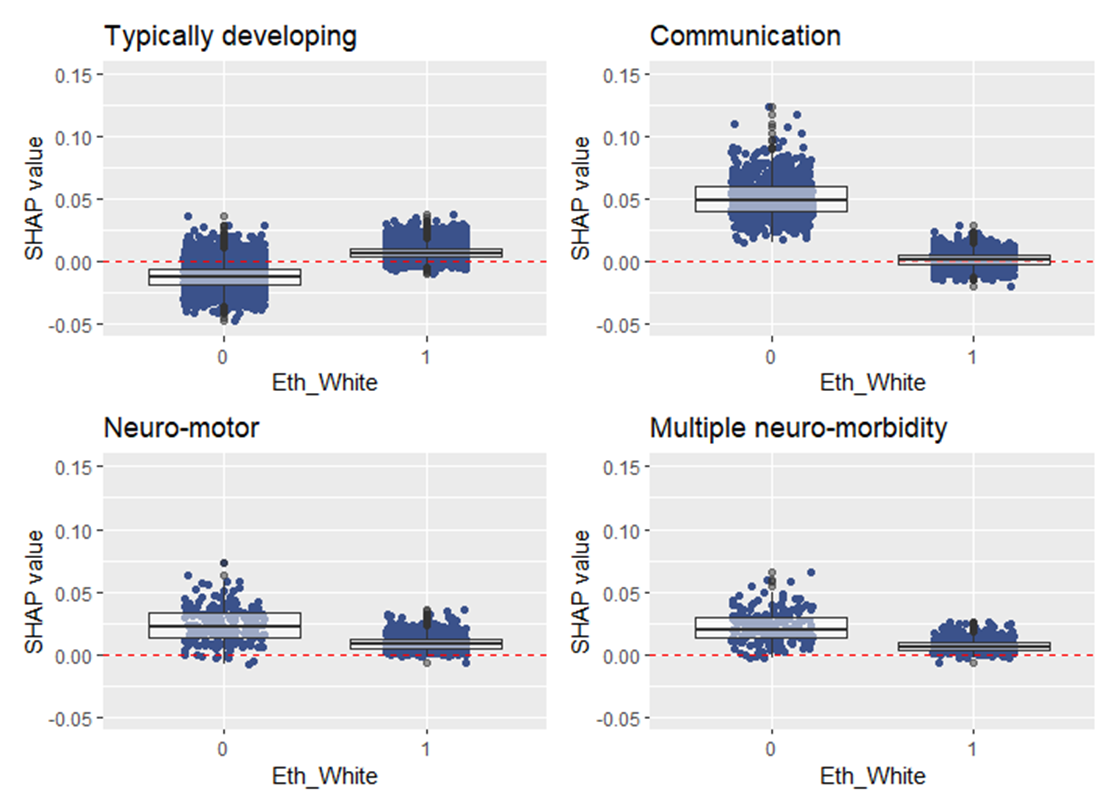


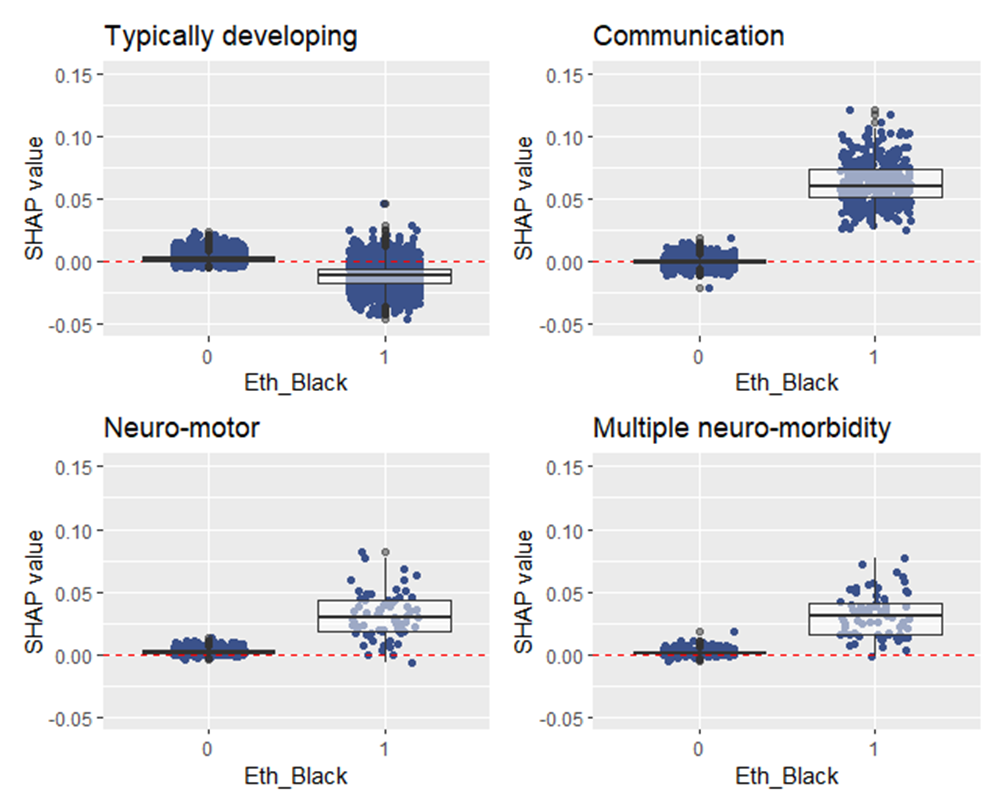


**
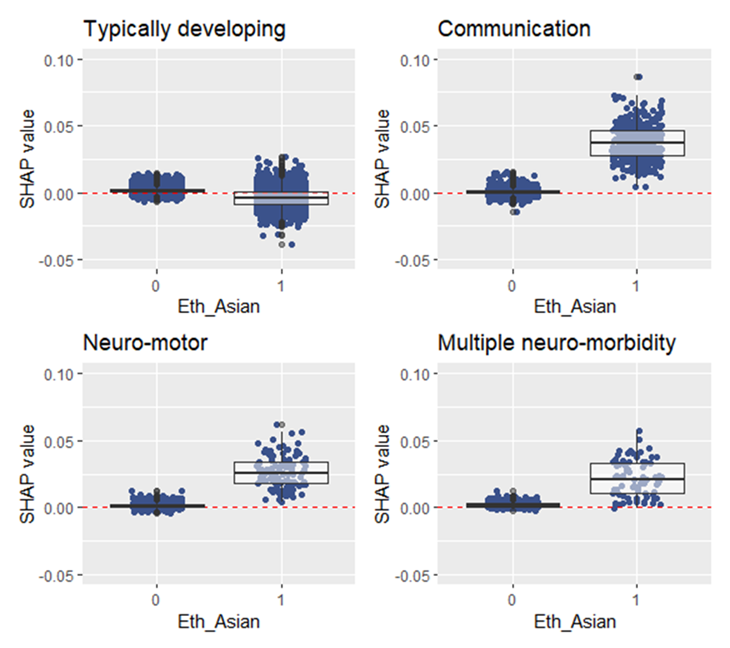
**

1. **Apgar score at 5 minutes**


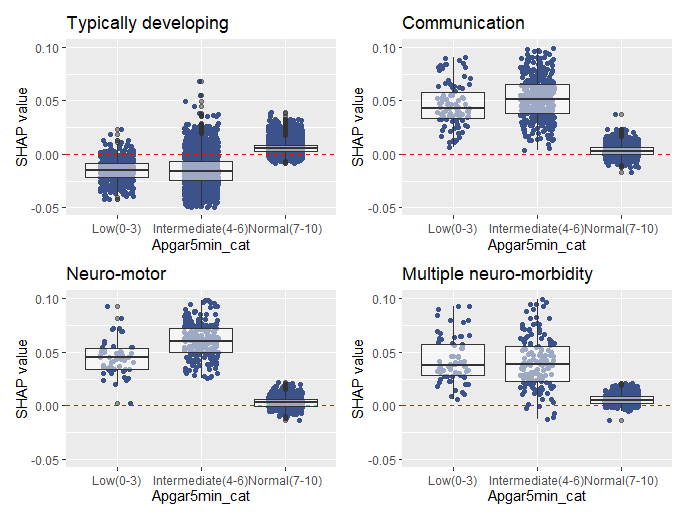


1. **Maternal age**

**
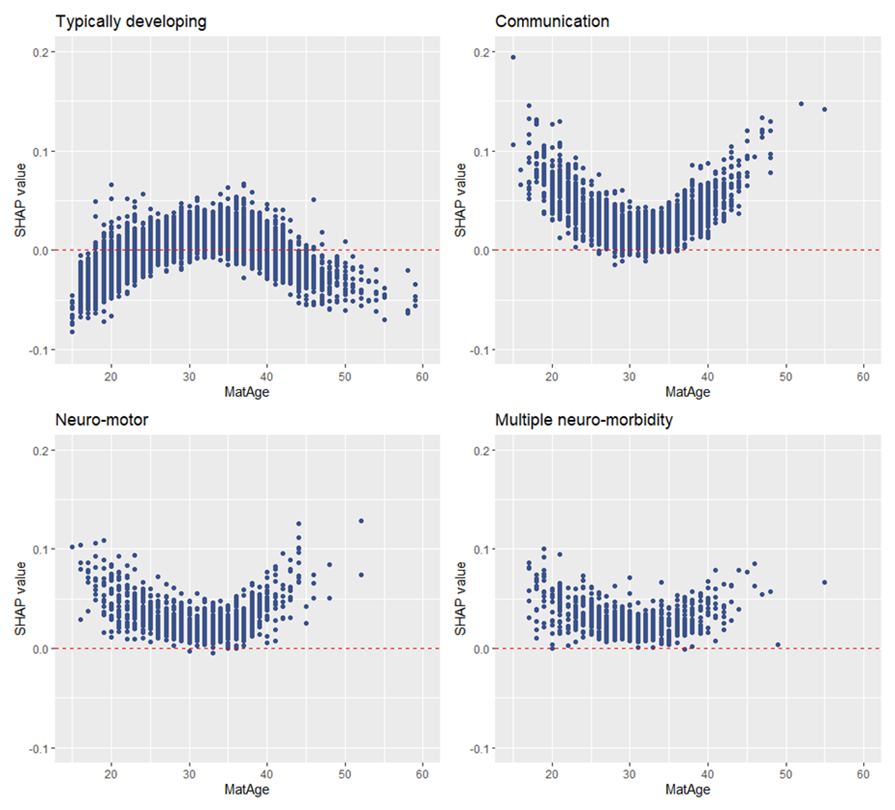
**

1. **Breast milk at discharge**

**
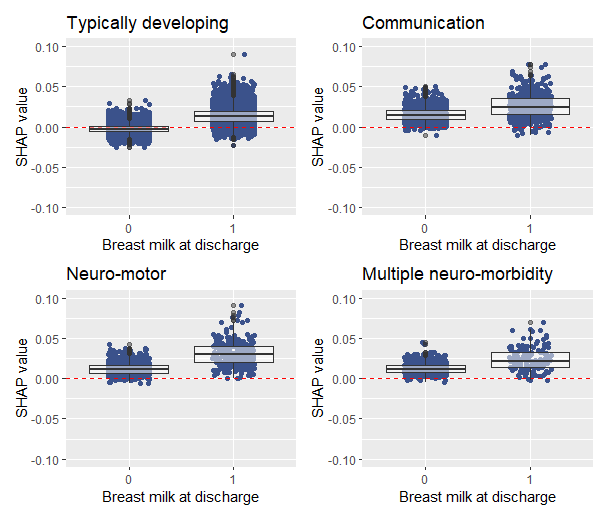
**

1. **Requirement for supplemental oxygen at discharge**


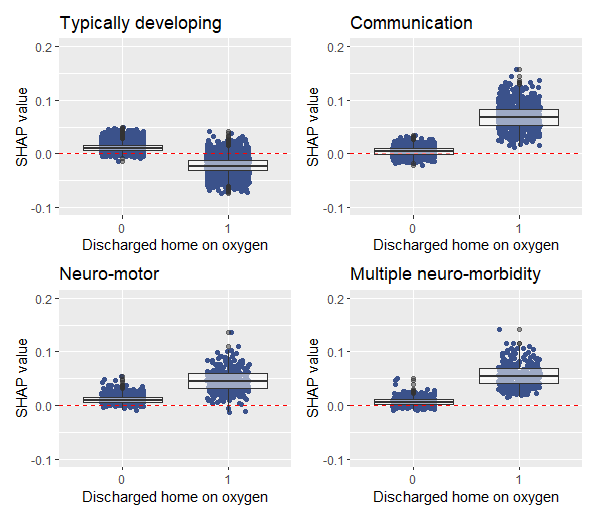


1. **Discharge destination**

**
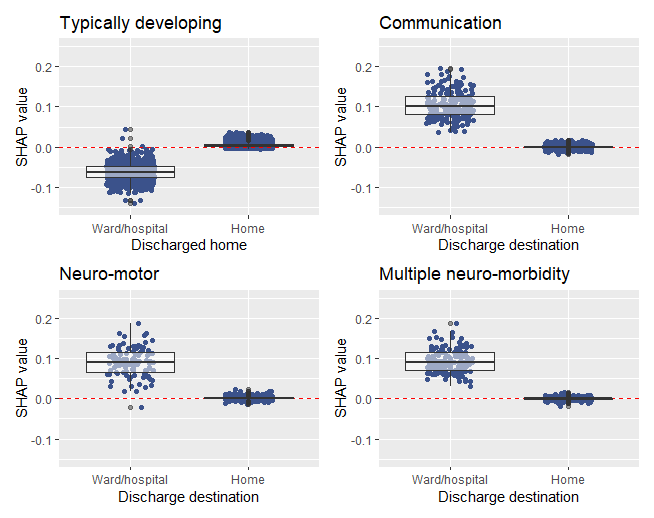
**

1. **Complete course of ante-natal steroids**

**
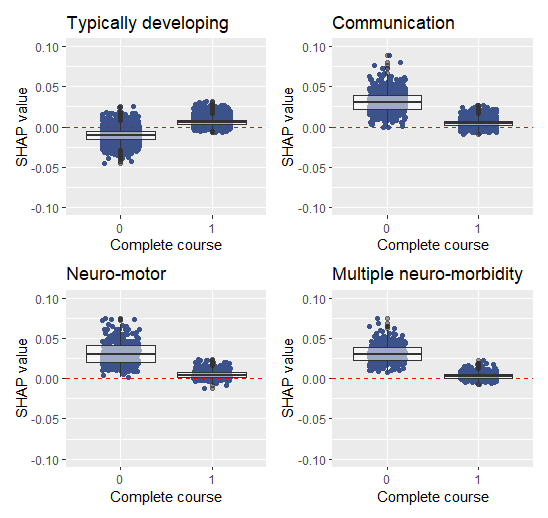
**

1. **Exposure to post-natal steroids**

**
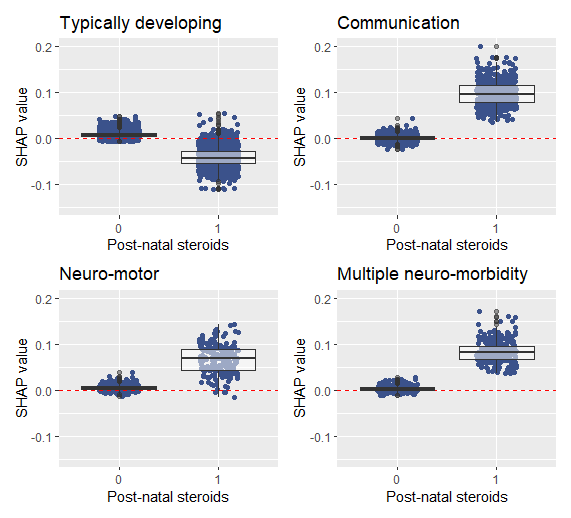
**

# Figure S9: Differences in random forest normalized feature importance scores between imputed and complete feature data

TD=Typically developing, COMM=Communication, NM=Neuro-motor, MNM=Multiple neuro-morbidity

**
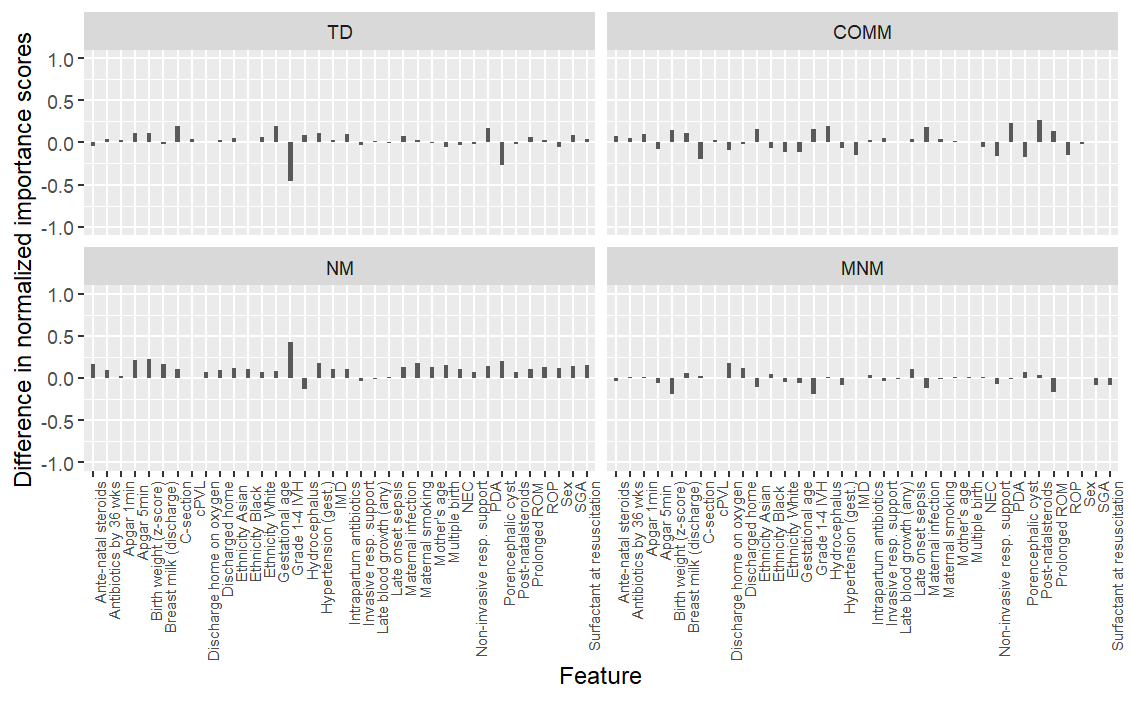
**

# References

1. Montgomery C, Setanen S, Kaul YF, Farooqi A, Brostrom L, Aden U, et al. Predictive value of Bayley-III Motor Index for later motor difficulties in children born extremely preterm. Acta Paediatr. 2023;112(4):742-52.
